# Gut Metabolite Indole‐3‐Propionic Acid Regulates Macrophage Autophagy Through PPT1 Inhibiting Aging‐Related Myocardial Fibrosis

**DOI:** 10.1002/advs.202501070

**Published:** 2025-06-20

**Authors:** Jing Lu, Hongyan Wang, Haiyu Zhang, Jiahao Li, Hanqi Li, Qiuyu Chen, Dongyu Han, Jialiang Liu, Lin Lv, Jie Xiong, Keying Yuan, Xianpeng Wei, Siqi Sheng, Fukai Liu, Yuanqi Shi, Zengxiang Dong, Yue Li

**Affiliations:** ^1^ Department of Pharmacy The First Affiliated Hospital of Harbin Medical University Harbin 150000 China; ^2^ The Key Laboratory of Cardiovascular Disease Acousto‐Optic Electromagnetic Diagnosis and Treatment in Heilongjiang Province The First Affiliated Hospital of Harbin Medical University Harbin 150000 China; ^3^ Animal Laboratory Center The First Affiliated Hospital of Harbin Medical University Harbin 150000 China; ^4^ Department of Cardiology The First Affiliated Hospital of Harbin Medical University Harbin 150000 China; ^5^ NHC Key Laboratory of Cell Transplantation The First Affiliated Hospital of Harbin Medical University Youzheng Street, Nangang District Harbin 150000 China

**Keywords:** aging‐related cardiac fibrosis, autophagy, IPA, macrophages, palmitoyl‐protein thioesterase 1

## Abstract

Cardiac fibrosis, a key pathological feature of cardiac remodeling, is a major contributor to mortality in older patients with heart failure. The underlying mechanisms are complex, involving alterations in intercellular communication and chronic inflammation. This study investigates the role of indole‐3‐propionic acid (IPA) in aging‐related myocardial fibrosis and its regulatory effects on autophagy through palmitoyl‐protein thioesterase 1 (PPT1). Here, plasma levels of IPA, a tryptophan‐derived metabolite, are found to be reduced in older patients with heart failure, and this reduction is associated with deteriorating cardiac function. Notably, IPA supplementation significantly attenuated aging‐related myocardial fibrosis. PPT1, a lysosomal enzyme involved in autophagy, is upregulated in macrophages during aging. IPA reversed aging‐induced increase in PPT1 expression. Using PPT1^flox/flox^ Lyz2‐cre mice, it is demonstrated that macrophage‐specific deletion of PPT1 significantly reduced cardiac inflammation and myocardial fibrosis in aged mice. Furthermore, PPT1 silencing in macrophages reduced the expression of myocardial fibrosis markers in vitro. Mechanistically, IPA regulated PPT1 expression to modulate the PI3K‐AKT‐mTOR pathway, thereby restoring autophagic activity in senescent macrophages and suppressing both inflammation and aging‐related myocardial fibrosis. Additionally, IPA influenced the cGAS‐STING signaling pathway to regulate PPT1 expression. These findings demonstrate that IPA inhibits PPT1, activates autophagy in macrophages, and mitigates aging‐related myocardial fibrosis.

## Graphical abstract

IPA is an intestinal tryptophan metabolite whose effects decline with decreased heart function. Supplementing IPA can alleviate the aging‐related myocardial fibrosis through PPT1. PPT1 is a key protein localized to lysosomes, and IPA can restore macrophage autophagy function by regulating PPT1 expresssions, thereby reducing aging‐related myocardial fibrosis and inflammation.

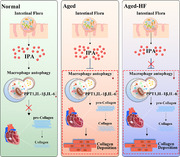

## 1 Introduction

Heart failure (HF) remains a leading cause of morbidity and mortality worldwide, with its incidence and prevalence increasing consistently.^[^ ^1^ ^]^ Pathological remodeling processes lead to phenotypic changes in cardiac fibroblasts, exacerbating extracellular matrix deposition. This excessive matrix accumulation reduces cardiac tissue compliance and ultimately contributes to the progression of advanced HF.^[^ ^2^ ^]^ Aging is a significant risk factor for myocardial fibrosis, playing a crucial role in its development and progression.^[^ ^3^ ^]^ However, the precise mechanisms underlying aging‐related myocardial fibrosis have not been fully elucidated. Consequently, identifying novel therapeutic targets is essential for developing effective interventions. Emerging evidence suggests that alterations in gut microbiota significantly influence the pathophysiology of cardiovascular diseases.^[^ ^4^, ^5^ ^]^ To facilitate genetic and molecular analyses, we utilized a rodent model of aging. Our findings revealed a reduction in circulating and cardiac tissue levels of indole‐3‐propionic acid (IPA) during aging, a gut‐derived metabolite synthesized from tryptophan by *Clostridium sporogenes*.^[^ ^6^ ^]^ Tryptophan, an essential amino acid, plays a key role in maintaining physiological equilibrium. IPA has been shown to regulate mitochondrial activity and modulate cardiac function.^[^ ^7^ ^]^ Additionally, it has demonstrated cardioprotective effects by preserving ejection fraction.^[^ ^8^ ^]^ However, whether IPA inhibits myocardial fibrosis in older individuals remains unclear.

Owing to the aging of the immune system, older individuals frequently experience low‐grade chronic inflammation, which is associated with an increased risk of cardiovascular disease.^[^ ^9^ ^]^ Studies have demonstrated a positive correlation between age and the number of cardiac macrophages.^[^ ^10^, ^11^ ^]^ In aging mice, the number of resident cardiac macrophages declines while the contribution of circulating blood monocytes to cardiac macrophage populations increases.^[^ ^12^ ^]^ Macrophages play a central role in promoting inflammation by secreting proinflammatory molecules such as interleukin (IL)‐6, tumor necrosis factor (TNF)‐α, and matrix metalloproteinases (MMPs), which collectively contribute to cardiac dysfunction.^[^ ^13^, ^14^ ^]^ Our study investigated macrophage‐mediated effects on the heart to identify potential therapeutic targets.

Palmitoyl‐protein thioesterase 1 (PPT1) is mainly located within lysosomes, where it regulates the degradation and clearance of lysosomal hydrolases, thereby influencing autophagy.^[^ ^15^, ^16^ ^]^ PPT1 plays a critical role in disease pathogenesis, as it modulates the lysosomal degradation of nucleotide‐binding domain‐like receptor protein 3 (NLRP3). By reducing the degradation of NLRP3, PPT1 enhances its activation, leading to the release of inflammatory factors and ultimately triggering the proinflammatory phenotype of macrophages.^[^ ^17^ ^]^ Notably, genetic defects in PPT1 or pharmacologic inhibition of PPT1 have been shown to reduce autoantibody levels and attenuate systemic inflammation.^[^ ^18^ ^]^ PPT1 is a target of transcription factor EB (TFEB), the master regulator of autophagy and lysosomal biogenesis, which orchestrates lysosomal function via PPT1 regulation.^[^ ^19^ ^]^ Therefore, we utilized PPT1^flox/flox^ Lyz2‐cre mice to examine the regulatory effects of PPT1 in the context of cardiovascular aging. In addition, we explored the effect of IPA on reducing inflammation and fibrosis in the aged model by regulating PPT1. This is the first study to investigate the role of PPT1 in aging‐related cardiovascular diseases.

We further explored the interaction of IPA with PPT1 by PI3K‐AKT‐mTOR signaling pathway. Activation of mTOR complex 1 (mTORC1) has been implicated in the proliferation and expansion of inflammatory monocytes, thereby exacerbating systemic inflammation.^[^ ^20^ ^]^ Conversely, pharmacologic inhibition of mTORC1 with rapamycin has been shown to mitigate inflammatory responses.^[^ ^21^ ^]^ The cyclic GMP‐AMP synthase (cGAS)‐stimulator of interferon genes (STING) pathway is a critical danger‐sensing mechanism involved in innate immunity.^[^ ^22^ ^]^ We hypothesize that IPA modulates PPT1 through the regulation of the cGAS‐STING pathway. STING activation in cardiomyocytes triggers downstream signaling cascades, leading to increased production of pro‐inflammatory cytokines and transforming growth factor‐beta (TGF‐β), which ultimately contribute to myocardial fibrosis.^[^ ^23^ ^]^ In this study, we investigated the specific mechanism by which IPA inhibits PPT1 activation and induces autophagy.

Our findings indicate that IPA is downregulated in older patients with HF and that IPA supplementation reduces the degree of myocardial fibrosis in aged rats, suggesting its potential as a therapeutic agent for aging‐related myocardial fibrosis. Moreover, macrophages highly expressing PPT1 exhibit a pro‐inflammatory phenotype significantly upregulated in senescent macrophages. Furthermore, IPA regulates macrophage PPT1 via the cGAS‐STING pathway while simultaneously modulating the autophagic function of macrophages through the PI3K‐AKT‐mTOR pathway.

## 2 Results

### 2.1 Abnormal Expression of Tryptophan and its Metabolite IPA in Aged Patients and Rats

**Figure 1:**
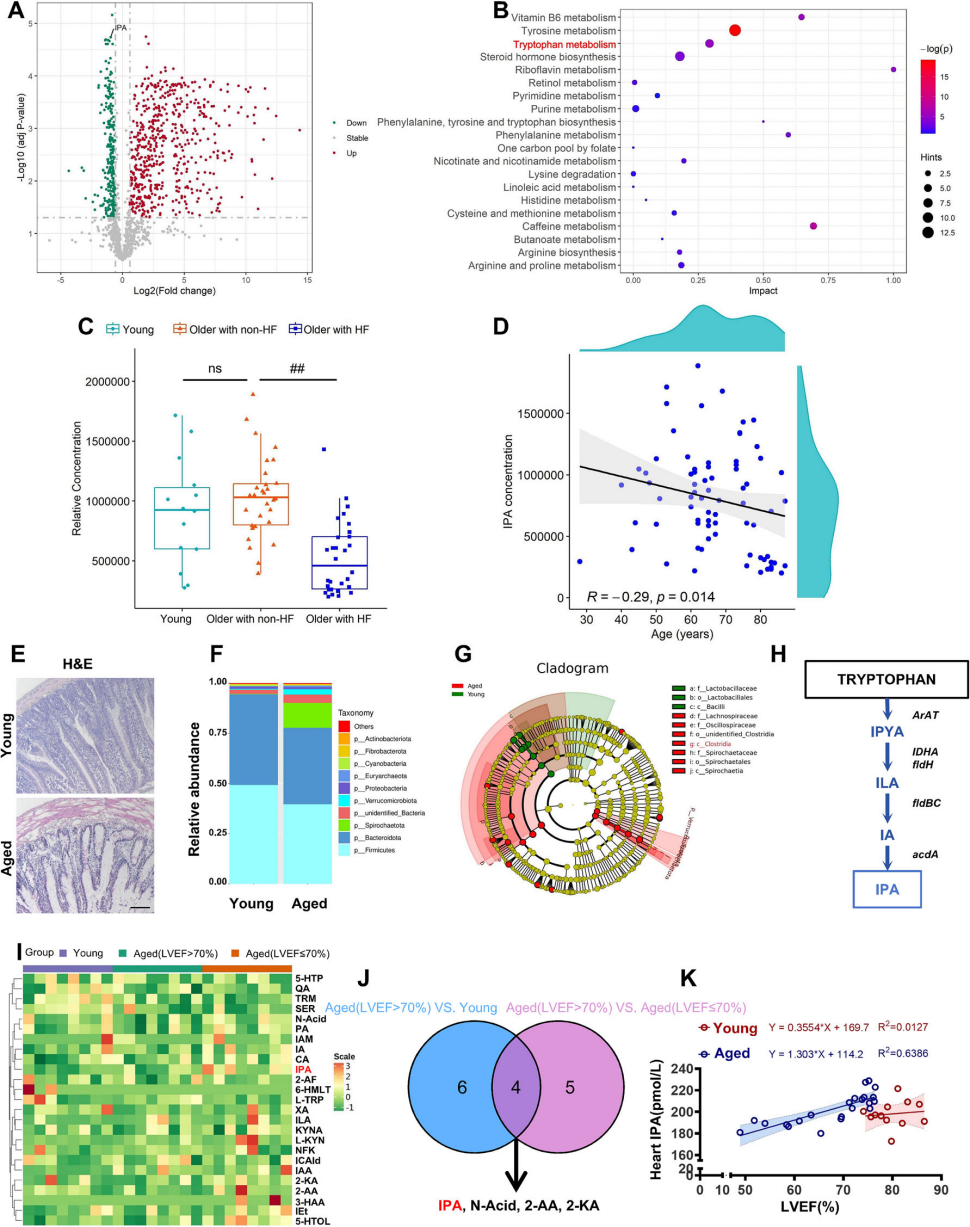
Abnormal expression of tryptophan and its metabolite IPA in tissue samples from elderly patients and rats. A) Volcano plot of differential metabolites in Older with non‐HF vs Older with HF groups of human plasma samples. B) KEGG pathway enrichment map of differential metabolites in Older with non‐HF vs Older with HF groups of human plasma samples. C) Box plots of IPA content in Young (*n* = 14), Older with non‐HF (*n* = 30) and Older with HF (*n* = 30) groups of human plasma samples (^##^ *p* < 0.01 vs the Older with non‐HF group). D) Correlation scatter plot between human plasma IPA concentration and age. E) H&E staining of rat colon samples in Young and Aged groups. (*n* = 3) Magnification: 100×, scale bar = 100 µm. F) Stacked bar plots of relative abundance of species at phylum level based on ASV of rat feces samples in Young (*n* = 8) and Aged (*n* = 8) groups. G) ASV‐based cladogram of rat feces samples in Young and Aged groups. H) Metabolic pathway of IPA in the tryptophan metabolic pathway of rat feces samples. I) Heat map of differential metabolites of rat feces samples in Young (*n* = 8), Aged (LVEF>70%) (*n* = 8), and Aged (LVEF≤70%) (*n* = 8) groups. J) Venn plot of differential metabolites of rat feces samples in Young, Aged (LVEF>70%), and Aged (LVEF≤70%) groups. K) Correlation scatter plot about rat heart IPA content and LVEF(%) value in Young (*n* = 12) and Aged (*n* = 24) group.

To evaluate plasma metabolite expression in older patients with heart failure, we performed metabolomic analysis on plasma samples from young (*n* = 14), older with non‐HF (*n* = 30), and older patients with heart failure (Older with HF, *n* = 30) (Table S1, Supporting Information). A metabolite difference volcano plot (**Figure** **1**A) and Kyoto Encyclopedia of Genes and Genomes (KEGG) pathway enrichment analysis revealed differential metabolites in human plasma, with tryptophan metabolism being significantly enriched in the older with HF group compared to the older with non‐HF group (Figure 1B). IPA was significantly downregulated in older with HF group compared with older with non‐HF group (Figure 1C). Correlation analysis revealed a negative correlation between IPA concentration and age (Figure 1D). Hematoxylin and eosin (H&E) staining revealed increased inflammation in the colons of the aged group (Figure 1E). To investigate gut microbiota composition, we collected and analyzed rat fecal samples using 16S rDNA sequencing. As shown in Figure 1F, the *Firmicutes/Bacteroidota* ratio was decreased in aged rats, indicating increased inflammation. Linear discriminant analysis effect size (LEfSe) analysis identified a significant difference in the relative abundance of *c_Clostridia* between groups (Figure 1G). Previous studies have shown that *c_Clostridium* is the primary bacteria responsible for metabolizing tryptophan into IPA (Figure 1H).^[^ ^24^ ^]^ Next, we performed targeted quantitative metabolomic analysis of rat fecal samples to measure IPA levels (Figure 1I). Venn diagram revealed that IPA, nicotinic acid (N‐acid), 2‐Aminobenzoic acid (2‐AA), and 2‐Ketoadipic acid (2‐KA) were common differentially expressed metabolites (Figure 1J). Furthermore, IPA levels in heart tissues were downregulated with left ventricular ejection fraction (LVEF) value (Figure 1K). These findings indicate that gut microbiota composition is altered in aged rats with heart failure and that IPA, a key tryptophan metabolite, is significantly reduced in plasma and heart tissue with aging and deterioration cardiac function.

### 2.2 IPA Inhibits the Secretion of Pro‐Inflammatory Factors in Senescent Macrophages and Alleviates Myocardial Fibrosis in Aged Rats

Aged rat models were established using 18‐ to 20‐month‐old Sprague‐Dawley (SD) rats (Figure S1A, Supporting Information). Echocardiography was performed to assess cardiac function (Figure S1B, Supporting Information), measuring LVEF, left ventricular fractional shortening (LVFS), left ventricular internal diameter at diastole (LVIDd), left ventricular internal diameter at systole (LVIDs), left ventricular posterior wall thickness at diastole (LVPWd), left ventricular posterior wall thickness at systole (LVPWs), interventricular septal end‐diastole (IVSd), and interventricular septal end‐systole (IVSs) were detected (Figure S1C–J, Supporting Information). A significant decline in cardiac function was observed in aged rats. H&E and Masson's staining revealed disordered cardiac muscle fibers and increased myocardial fibrosis in aged hearts (Figure S1K–M, Supporting Information). Additionally, aged rats exhibited an upregulation of senescence markers P21 and P16, along with increased levels of fibrosis‐related genes COL1A1 and COL3A1 and pro‐inflammatory markers IL‐6 and CD86 in aged rats (Figure S1N,O, Supporting Information). These findings confirm that aging leads to significant cardiac dysfunction and myocardial fibrosis.

**Figure 2:**
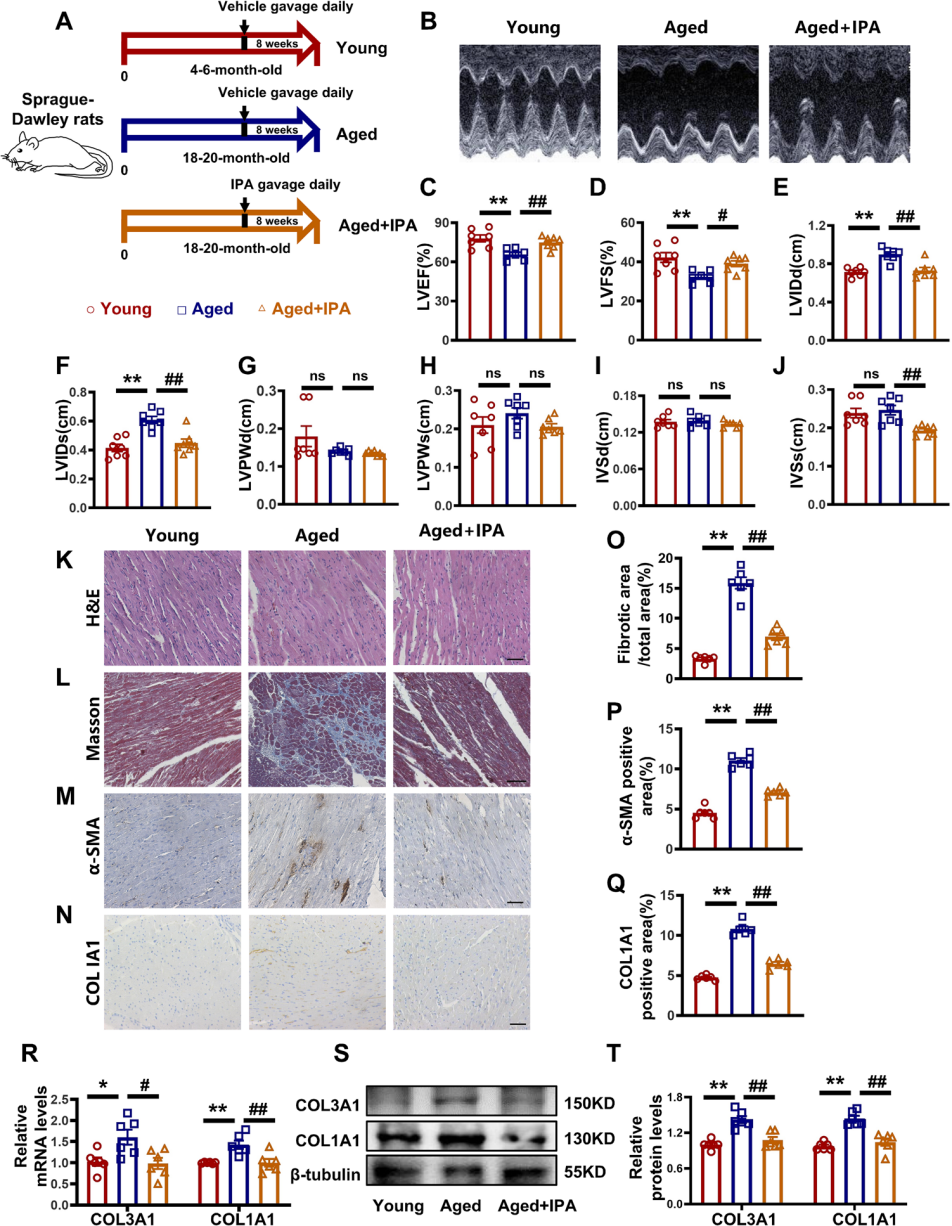
IPA alleviates aging‐related myocardial fibrosis in rats. A) Schematic of the experimental design. B) Representative echocardiographic graphs. C–J) Echocardiographic measurements of LVEF, LVFS, LVIDd, LVIDs, LVPWd, LVPWs, IVSd, and IVSs. K) H&E staining of rat hearts. Magnification: 200×, scale bar=50 µm. L) Masson's staining of rat hearts. Magnification: 100×, scale bar = 100 µm. M,N) Representative images of IHC staining with α‐SMA or COL1A1 antibody. Magnification: 200×, scale bar = 50 µm. O) Statistical graphs of Masson's staining. P,Q) Statistical graphs of IHC staining with α‐SMA or COL1A1. R) Statistical graphs of RT‐qPCR of COL3A1 and COL1A1. S,T) Representative images and statistical graphs of COL3A1 and COL1A1 at protein levels. (*n* = 6–7, data are expressed as mean ± SEM, ^*^ *p <* 0.05, ^**^ *p <* 0.01 vs the Young group; ^#^ *p <* 0.05, ^##^ *p <* 0.01 vs the Aged group).

To investigate the therapeutic potential of IPA in aging‐related myocardial fibrosis, aged rats were administered IPA (20 mg kg^−1^ day^−1^) by oral gavage for 8 weeks (**Figure** **2**A). Echocardiography was used to monitor cardiac function (Figure 2B), with additional cardiac function indices measured (Figure 2C–J). Histological evaluation using H&E and Masson's staining demonstrated that IPA treatment mitigated myocardial fibrosis and improved cardiac muscle fiber organization (Figure 2K,L, Figure 2O). Immunohistochemistry (IHC) analysis using α‐SMA and COL1A1 antibodies revealed that IPA significantly reduced α‐SMA and COL1A1‐positive areas in aged heart tissue (Figure 2M,N,P,Q). COL3A1 and COL1A1 were significantly reduced in the IPA‐treated aged group (Figure 2R–T). The detection of the IPA concentration in the heart after intragastric administration is shown in Figure S2A (Supporting Information). These findings suggest that IPA attenuates aging‐related myocardial fibrosis in aged rats.

**Figure 3:**
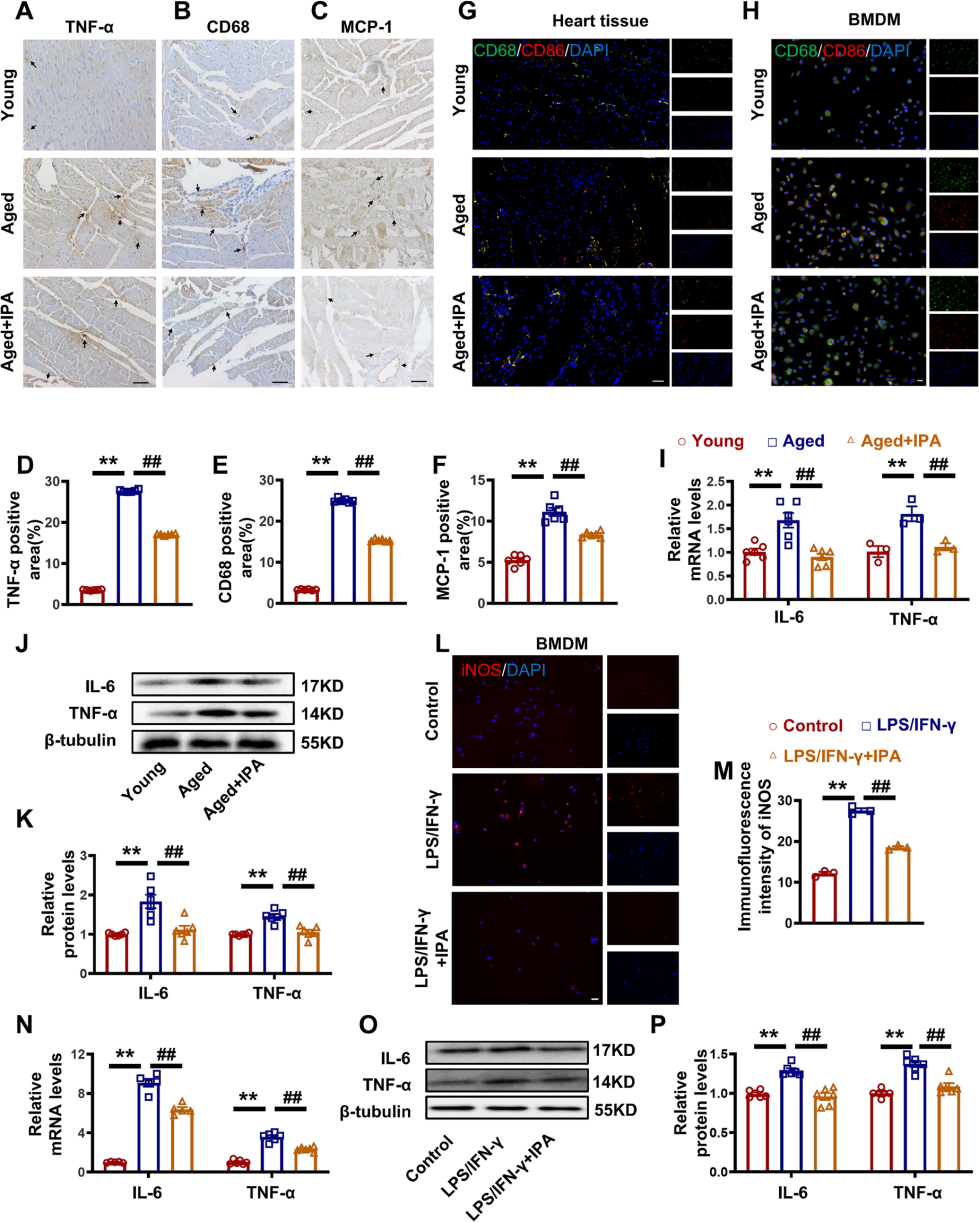
IPA inhibits proinflammatory factor secretion in senescent macrophages. A–F) Representative images and statistical graphs of IHC staining with TNF‐α, CD68 and MCP‐1 antibody. Magnification: 200×, scale bar = 50 µm. G) Representative images of the immunofluorescence of CD68, CD86, and DAPI in rat heart tissue (CD68^+^CD86^+^ represent M1 macrophage). Magnification: 200×, scale bar = 20 µm. H) Representative images of the immunofluorescence of CD68, CD86, and DAPI in rat BMDM. Magnification: 200×, scale bar = 20 µm. I) Statistical graphs showing RT‐qPCR results of IL‐6 and TNF‐α expression of rat hearts from the Young, Aged, and Aged+IPA groups. J,K) Representative images and statistical graphs of IL‐6 and TNF‐α at protein levels. L,M) Representative graphs of the immunofluorescence of iNOS in Control, LPS/IFN‐γ and LPS/IFN‐γ+IPA groups. Magnification: 200×, scale bar = 20 µm. N) Statistical graphs of RT‐qPCR of IL‐6 and TNF‐α in Control, LPS/IFN‐γ and LPS/IFN‐γ+IPA groups. O,P) Representative images and statistical graphs of IL‐6 and TNF‐α at protein levels in Control, LPS/IFN‐γ and LPS/IFN‐γ+IPA groups. (*n* = 3–6, data are expressed as mean ± SEM, ^**^ *p <* 0.01 vs the Young or Control group; ^##^ *p <* 0.01 vs the Aged or LPS/IFN‐γ group).

To determine the specific cell types through which IPA exerts its cardioprotective effects, primary neonatal rat cardiomyocytes and cardiac fibroblasts were isolated and cultured. Cellular senescence was induced by treating these cells with 50 µm hydrogen peroxide (H_2_O_2_) for 48h (Figure 2B–E). CIBERSORT analysis revealed compositional changes in immune cells within the ventricles of aged mice (Figure S2F–H, Supporting Information). As shown in **Figure** **3**A–F, the TNF‐α, CD68, and MCP‐1 positive areas were significantly reduced in the heart tissue of the Aged+IPA group, whereas these markers were notably increased in the Aged group. Immunofluorescence (IF) analysis further demonstrated M1 macrophages (CD68^+^CD86^+^) accumulated in the heart and bone marrow‐derived macrophages (BMDMs) with aging, but their numbers significantly decreased following IPA treatment (Figure 3G,H). Additionally, levels of IL‐6 and TNF‐α were increased in the Aged group, but IPA administration effectively reversed these increases (Figure 3I–K). Furthermore, IF staining showed a reduction in the intensity of iNOS in vitro (Figure 3L,M). To further confirm the anti‐inflammatory effects of IPA, we evaluated the expression of IL‐6 and TNF‐α (Figure 3N–P). These findings suggest that IPA inhibits M1 macrophage polarization, suppresses the expression of inflammatory cytokines, and exerts a protective effect against cardiac fibrosis and inflammation in aging.

### 2.3 Knockout of Macrophage PPT1 can Reduce Cardiac Inflammatory Infiltration and Myocardial Fibrosis in Aging Mice

**Figure 4:**
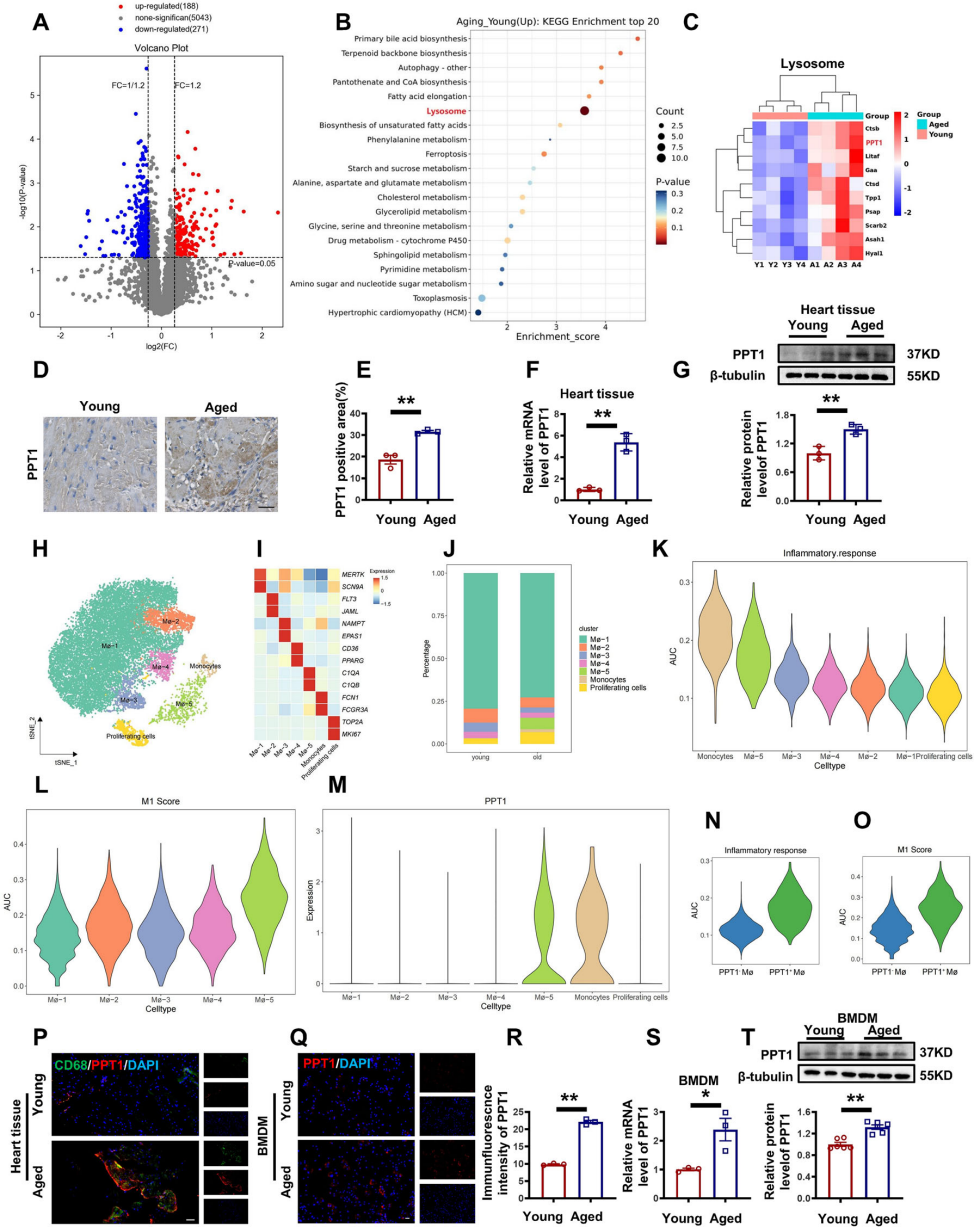
PPT1 expression is upregulated in senescent macrophages. A) Volcano plot of differentially expressed proteins of Young and Aged groups in rats heart. B) KEGG enrichment top 20. C) Heat map of differentially expressed proteins in the lysosomal pathway. D,E) Representative images and statistical analysis of IHC staining with PPT1 antibody. Magnification: 400×, scale bar = 100 µm. F) Statistical analysis of PPT1 mRNA level. G) Representative images and statistical analysis of PPT1 protein level. H) UMAP plots of heart macrophage from 27 healthy donors. I) A heatmap showing the markers ≈7 types of macrophages. J) Stacked graph of cell proportions. K) Violin plots of inflammatory response scores in different subgroups. L) Violin plots of the M1 scores of different macrophage subsets. M) Violin plots of PPT1 expression in different monocyte‐macrophage subsets. N) Inflammatory response score. O) M1 score. P) Representative figure of the immunofluorescence of CD68, PPT1, and DAPI in heart. Magnification: 200×, scale bar = 20 µm. Q,R) Representative immunofluorescence images and statistical analysis of PPT1 intensity. Magnification: 200×, scale bar = 20 µm. S) Statistical analysis of PPT1 mRNA level in BMDM. T) Representative graphs and statistical analysis of PPT1 at protein level in BMDM. (*n* = 3–6, data are expressed as mean ± SEM, ^*^ *p <* 0.05, ^**^ *p <* 0.01 vs the Young group).

Macrophages are the predominant resident immune cells in the heart and play a crucial role in maintaining cardiac homeostasis.^[^ ^25^ ^]^ To investigate macrophage alterations during aging, we performed flow cytometry analysis, which revealed a significant increase in the M1/M2 macrophage ratio (CD45^+^CD86^+^/ CD45^+^CD163^+^) in the Aged group (Figure S3A,B, Supporting Information). Both protein and mRNA levels of P21 and TNF‐α were markedly upregulated in the aged group (Figure S3C–F, Supporting Information), indicating enhanced inflammatory infiltration and a pro‐inflammatory phenotype in senescent macrophages. Proteomic sequencing revealed differences between the two groups (**Figure** **4**A). KEGG pathway analysis revealed alterations in the lysosomal pathway in aged hearts, with a significant upregulation of PPT1 expression (Figure 4B,C). PPT1 positive area, mRNA, and protein levels were increased in aged heart tissue (Figure 4D–G). To analyze the inflammatory response of PPT1 in macrophages, we analyzed single‐cell RNA sequencing (scRNA‐seq) data from 27 healthy donors.^[^ ^26^ ^]^ Monocytes and macrophages were classified into seven subsets (Figure 4H) and the marker is shown in Figure 4I. Percentage of 5 celltypes of macrophages is shown in Figure 4J. The monocyte subset had a higher immune response, as demonstrated by the box plot analysis (Figure 4K). The M1 score box plot further confirmed that the Mφ‐5 subset had the highest M1 polarization, with significantly elevated PPT1 expression in the Mφ‐5 and monocyte subsets—identifying them as highly inflammatory cell subsets (Figure 4L,M). Subsequently, we found that PPT1^+^ mice exhibited heightened immune responses and increased M1 scores (Figure 4N,O). IF staining confirmed a significant upregulation of PPT1 expression in CD68^+^ macrophages in aged hearts and BMDMs (Figure 4P–R). Similarly, PPT1 levels were upregulated in aged macrophages (Figure 4S,T). Collectively, these findings indicate that aging is associated with an increased proportion of pro‐inflammatory monocytes and macrophages, with pro‐inflammatory PPT1^+^ cells highly expressed in the heart.

**Figure 5:**
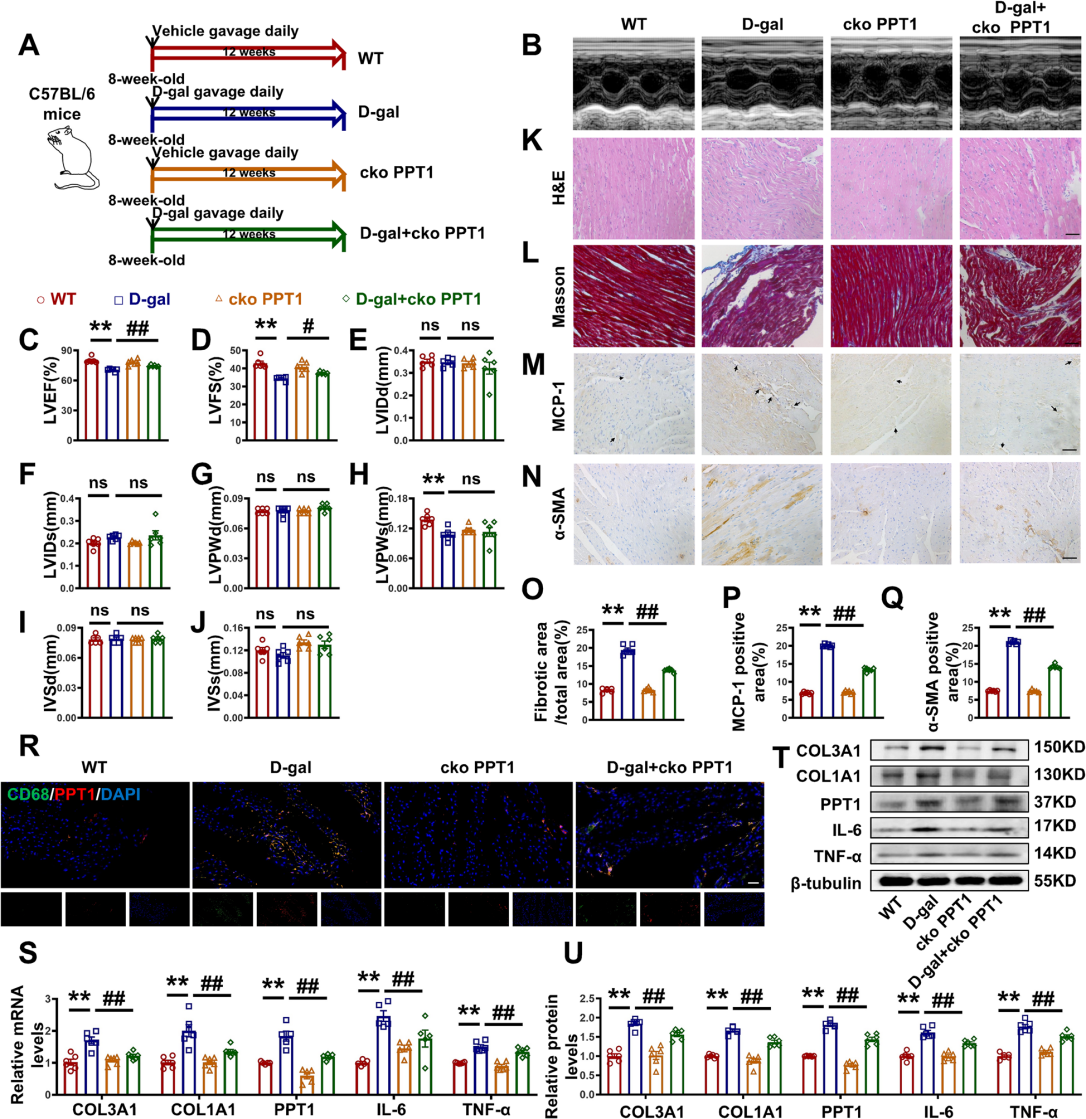
Transgenic knockout of macrophage PPT1 improves cardiac inflammatory infiltration and myocardial fibrosis in D‐gal induce‐aged mice. A) Schematic of the experimental design. B) Representative echocardiographic graphs. C–J) Echocardiographic measurements of LVEF, LVFS, LVIDd, LVIDs, LVPWd, LVPWs, IVSd, and IVSs. K) H&E staining of left ventricles. Magnification: 200×, scale bar = 50 µm. L) Masson's staining of left ventricles. Magnification: 200×, scale bar = 50 µm. M,N) Representative images of IHC staining with MCP‐1 and α‐SMA antibody. Magnification: 200×, scale bar = 50 µm. O) Statistical graphs of Masson's staining. P,Q) Statistical graphs of IHC staining with MCP‐1 and α‐SMA antibody. R) Representative images of the immunofluorescence of CD68, PPT1, and DAPI in mice heart. Magnification: 200×, scale bar = 20 µm. S) Statistical analysis of COL3A1, COL1A1, PPT1, IL‐6, and TNF‐α mRNA level. T,U) Representative images and statistical analysis of COL3A1, COL1A1, PPT1, IL‐6, and TNF‐α at protein level. (*n* = 5–6, data are expressed as mean ± SEM, ^**^ *p <* 0.01 vs the WT group; ^#^ *p <* 0.05, ^##^ *p <* 0.01 vs the D‐gal group).

To verify the role of PPT1 in the heart during aging, PPT1^flox/flox^ Lyz2‐cre mice (cko‐PPT1) were administered D‐galactose (D‐gal) for 12 weeks to induce aging (**Figure** **5**A). In addition, adeno‐associated virus (AAV)‐F4/80‐sh‐PPT1 was injected into the aged rats to induce PPT1 knockdown (Figure S4A, Supporting Information). Echocardiography was performed to assess cardiac function (Figure 5B; Figure S4B, Supporting Information). The results showed that PPT1 knockdown via either cko‐PPT1 or sh‐PPT1 attenuated the decline in cardiac function induced by D‐gal or aging (Figure 5C–J; Figure S4C–J, Supporting Information). H&E and Masson's staining revealed that cko‐PPT1 or sh‐PPT1 reversed the inflammation, disordered muscle fibers, and excessive collagen deposition induced by D‐gal or aging (Figure 5K,L,O; Figure S4K,L,O, Supporting Information). IHC further demonstrated that cko‐PPT1 or sh‐PPT1 reduced the D‐gal‐ or aging‐induced MCP‐1 and α‐SMA positive areas (Figure 5M,N,P,Q; Figure S4M,N,P,Q, Supporting Information). Additionally, IF staining showed that macrophage PPT1 (CD68^+^PPT1^+^) expression increased with aging, while cko‐PPT1 or sh‐PPT1 decreased the number of CD68^+^PPT1^+^ cells (Figure 5R; Figure S4R, Supporting Information). Fibrotic and inflammatory markers were also downregulated in the D‐gal + cko‐PPT1 and aging + sh‐PPT1 groups (Figure 5S–U; Figure S4S–U, Supporting Information). These findings indicate that cko‐PPT1 or sh‐PPT1 could reverse the aging‐induced increase in fibrosis and inflammation.

### 2.4 IPA Regulates PPT1 to Inhibit the Secretion of Pro‐Inflammatory Factors in Senescent Macrophages

To investigate the pro‐inflammatory effects of macrophage PPT1, we employed si‐PPT1 or DC661, a PPT1 inhibitor, and found that si‐PPT1#1 had a better knockdown effect (Figure S5A,B, Supporting Information). Subsequently, both si‐PPT1 and DC661 significantly reduced iNOS levels induced by LPS/IFN‐γ in vitro (Figures S5C,D and S6A,B, Supporting Information). Furthermore, we examined the impact of PPT1 knockdown on M1 macrophages using si‐PPT1 or DC661 and observed a significant reduction in their pro‐inflammatory activity (Figures S5E,F and S6C–E, Supporting Information). These findings suggest that the knockdown of PPT1 could significantly inhibit the pro‐inflammatory effects of M1 macrophages.

To determine whether IPA mediates PPT1 regulation in macrophages, we assessed PPT1 expression in aged and M1 macrophages using IF and western blotting. Our results demonstrated that IPA reduced the expression of PPT1 both in vivo and in vitro (Figure S7A–E, Supporting Information). This effect was further validated by PPT1 overexpression (Figure S7F,G, Supporting Information), followed by IPA treatment, which confirmed that IPA could reduce PPT1 levels despite its overexpression (Figure S7H,I, Supporting Information).

**Figure 6:**
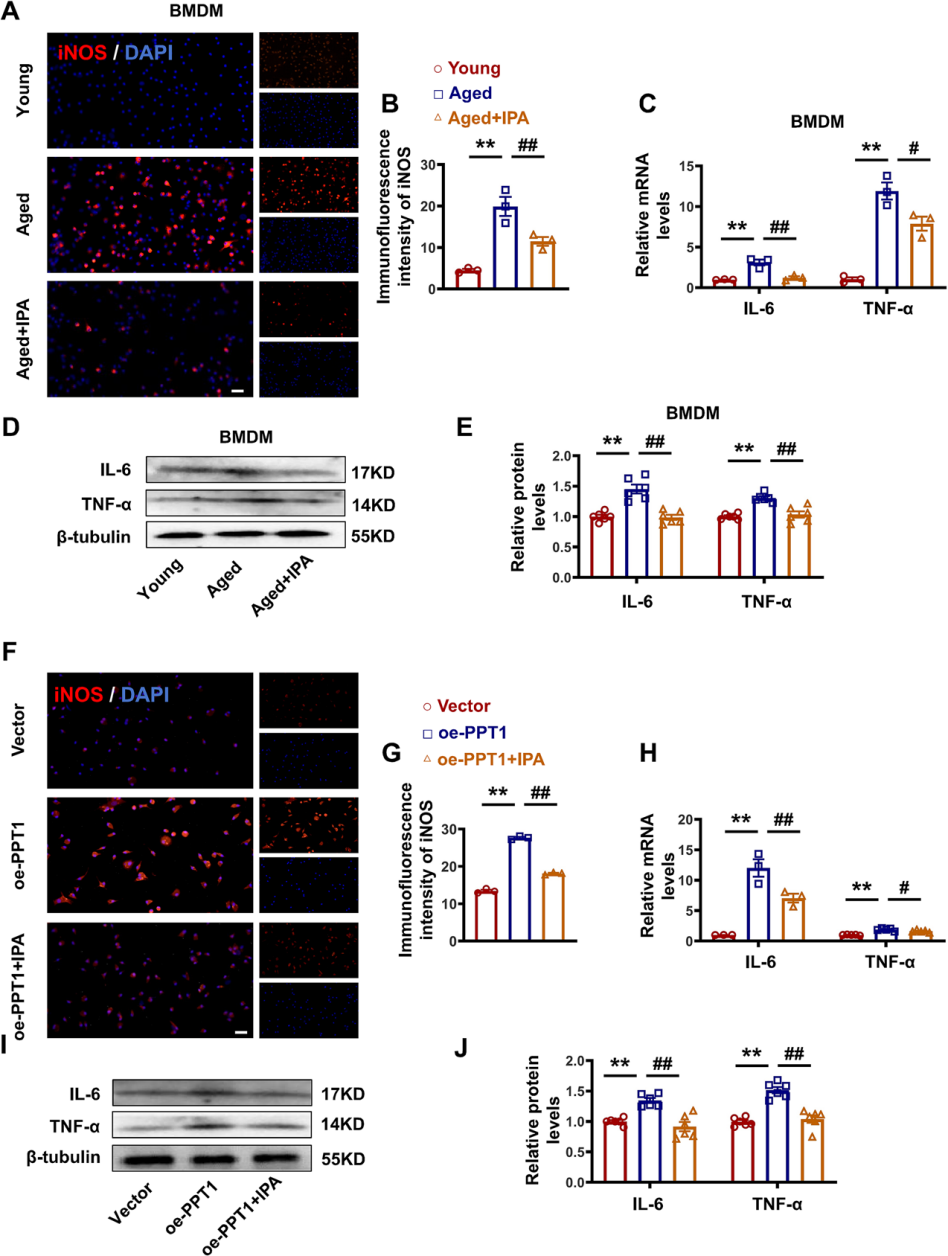
IPA inhibits PPT1 expression and reduces the secretion of inflammatory factors in aging macrophages. A,B) Representative images and statistical graphs of the immunofluorescence of iNOS. Magnification: 200×, scale bar = 20 µm. C) Statistical analysis of IL‐6 and TNF‐α at mRNA level. D,E) Representative images and statistical analysis of IL‐6 and TNF‐α at protein level. F,G) Representative images and statistical graphs of the immunofluorescence of iNOS. Magnification: 200×, scale bar = 20 µm. H) Statistical analysis of IL‐6 and TNF‐α at mRNA level. I,J) Representative images and statistical analysis of IL‐6 and TNF‐α at protein level. (*n* = 3–6, data are expressed as mean ± SEM, ^**^ *p <* 0.01 vs the Young or Vector group; ^#^ *p <* 0.05, ^##^ *p <* 0.01 vs the Aged or oe‐PPT1 group).

To further investigate whether IPA regulates senescent macrophages through PPT1, we first verified the anti‐inflammatory effects of IPA in aged macrophages, which showed a reduction in pro‐inflammatory marker levels (**Figure** **6**A–E). Similarly, we found that IPA attenuated the pro‐inflammatory effects of oe‐PPT1 (Figure 6F–J). Collectively, these results provide compelling evidence that IPA could regulate the inflammatory response in aged macrophages through PPT1.

### 2.5 IPA Regulates PPT1 to Inhibit Senescent Macrophages from Inducing Myocardial Fibrosis

A previous study reported that macrophage‐fibroblast crosstalk plays an important role in the pathophysiology of cardiac inflammation and fibrosis.^[^ ^27^ ^]^ To further investigate the role of PPT1‐expressing macrophages in myocardial fibrosis, conditioned medium (CM) from M1 macrophages was harvested and added to cardiac fibroblasts (Figure S8A, Supporting Information). BMDMs were extracted from young and aged rats, treated with si‐PPT1 or DC661, and their CM was collected and administered to cardiac fibroblasts. As shown in Figure S8B,C,F,G (Supporting Information), PPT1 inhibition via si‐PPT1 or DC661 significantly reduced α‐SMA expression in cardiac fibroblasts. Western blot analysis further showed that incubation of aged BMDMs with si‐PPT1 or DC661 alleviated fibrosis in cardiac fibroblasts by decreasing the expression of COL3A1, COL1A1, and α‐SMA (Figure S8D,E,H,I, Supporting Information). These findings indicate that inhibiting PPT1 in aged macrophages reduces collagen deposition in myocardial fibroblasts.

**Figure 7:**
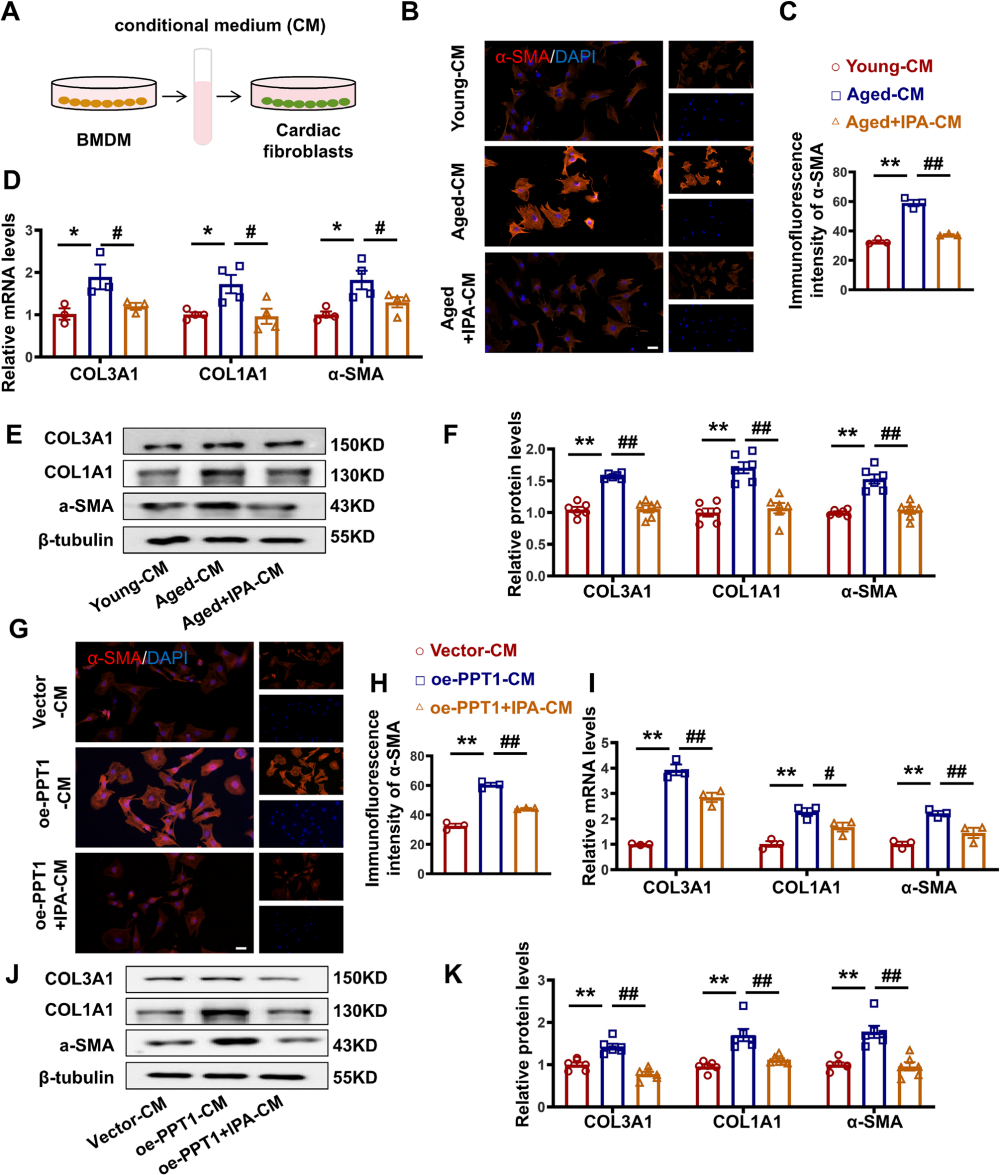
IPA inhibits PPT1 in aged macrophages and alleviates collagen deposition in fibroblasts. A) Schematic of the experimental design. B,C) Representative figures and statistical graphs of the immunofluorescence of α‐SMA. Magnification: 200×, scale bar = 20 µm. D) Statistical analysis of COL3A1, COL1A1 and α‐SMA at mRNA level. E,F) Representative images and statistical analysis of COL3A1, COL1A1 and α‐SMA at protein level. G,H) Representative Figure and statistical graphs of the immunofluorescence of α‐SMA. Magnification: 200×, scale bar = 20 µm. I) Statistical analysis of COL3A1, COL1A1 and α‐SMA at mRNA level. J,K) Representative figures and statistical analysis of COL3A1, COL1A1 and α‐SMA at protein level. (*n* = 3–6, data are expressed as mean ± SEM, ^*^ *p <* 0.5, ^**^ *p <* 0.01 vs the Young–CM or Vector–CM group; ^#^ *p <* 0.5, ^##^ *p <* 0.01 vs the Aged–CM or oe‐PPT1–CM group).

Next, we assessed whether IPA regulates myocardial fibrosis through its effects on PPT1 in the macrophages of aged rats. We measured fibrosis marker expression levels in cardiac fibroblasts which influenced by Young, Aged, and Aged + IPA macrophages and found that Aged + IPA‐CM reversed the Aged‐CM‐induced upregulation of α‐SMA (**Figure** **7**A–F). Similarly, IPA treatment reversed the oe‐PPT1‐induced increases in COL3A1, COL1A1, and α‐SMA levels (Figure 7G–K). These findings suggest that IPA regulates the crosstalk between macrophages and fibroblasts via PPT1, thereby attenuating fibroblast fibrosis.

**Figure 8:**
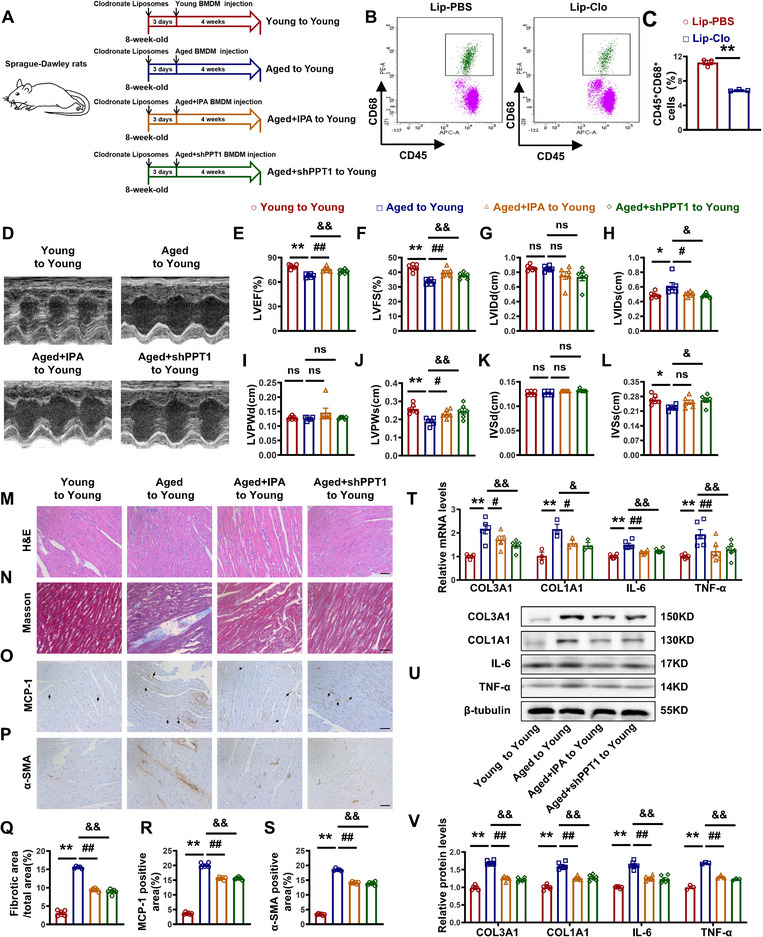
IPA inhibits PPT1 in elderly macrophages in vitro and reduces myocardial fibrosis in vivo. A) Schematic of the experimental design. B,C) Representative graphs and statistical graph of flow cytometry. D) Representative echocardiographic graphs. E–L) Echocardiographic measurements of LVEF, LVFS, LVIDd, LVIDs, LVPWd, LVPWs, IVSd, and IVSs. M) H&E staining of left ventricles. Magnification: 200×, scale bar = 50 µm. N) Masson's staining of left ventricles. Magnification: 200×, scale bar = 50 µm. O,P) Representative images of IHC staining with MCP‐1 and α‐SMA antibody. Magnification: 200×, scale bar = 50 µm. Q) Statistical graphs of Masson's staining. R,S) Statistical graphs of IHC staining with MCP‐1 and α‐SMA antibody. T) Statistical analysis of COL3A1, COL1A1, IL‐6, and TNF‐α at mRNA level. U,V) Representative images and statistical analysis of COL3A1, COL1A1, IL‐6, and TNF‐α at protein level. (*n* = 3–6, data are expressed as mean ± SEM, ^*^ *p <* 0.05, ^**^ *p <* 0.01 vs the Young group; ^#^ *p <* 0.05, ^##^ *p <* 0.01 vs the Aged group; ^&^ *p <* 0.05, ^&&^ *p <* 0.01 vs the Aged+sh‐PPT1 group).

While previous studies have established a correlation between macrophage dysfunction and aging‐related myocardial fibrosis, additional cell‐specific interventions, such as adoptive macrophage transfer experiments, are required to establish a causal relationship. To validate the regulatory effects of IPA on PPT1 expression in macrophages, we performed adoptive macrophage‐transfer experiments. Clodronate liposomes (Lip‐Clo) were used to selectively deplete macrophages in young rats. Macrophages from Young, Aged, Aged + IPA, and Aged + sh‐PPT1 rats were then transferred into young rats to assess cardiac function (**Figure** **8**A). Flow cytometry confirmed a significant reduction in macrophage levels in the Lip‐Clo group (Figure 8B,C). Echocardiography was performed to monitor cardiac function (Figure 8D), revealing that Aged + IPA or Aged + sh‐PPT1 macrophage transfer improved cardiac function compared with Aged macrophage transfer alone (Figure 8E–L). H&E and Masson's staining showed increased inflammation and collagen deposition in the Aged to the Young group, whereas Aged + IPA or Aged + sh‐PPT1 mitigated these effects (Figure 8M,N,Q). Furthermore, the MCP‐1 and α‐SMA‐positive areas were increased in the Aged to Young group, but both IPA and sh‐PPT1 treatment reduced these markers (Figure 8O,P,R,S). The mRNA and protein levels of fibrosis and inflammatory markers corroborated these findings (Figure 8T–V).

### 2.6 IPA Regulates Macrophage Autophagy Function Through PPT1

Macrophage autophagy decreases with age, leading to aggravated inflammation.^[^ ^28^ ^]^ Given the regulatory effect of IPA on autophagy and its impact on PPT1 expression in senescent macrophages, further investigation was warranted. To assess autophagic flux, BMDMs were infected with Ad‐GFP‐mCherry‐LC3B. Live‐cell imaging of BMDM showed spots with both GFP and mCherry fluorescence (i.e., autophagosomes) in the LPS/IFN‐γ group and spots with only mCherry fluorescence in the LPS/IFN‐γ+IPA group (i.e., autolysosomes) (Figure S9A, Supporting Information). Immunofluorescence analysis demonstrated that LC3B‐II formed distinct spots or clumps within the cell in both the control and LPS/IFN‐γ+IPA groups, indicative of normal autophagic activity (Figure S9B, Supporting Information). LC3B‐II and Beclin1 protein expression was upregulated, whereas P62 protein expression was downregulated (Figure S9C, Supporting Information). These data demonstrate that IPA activates autophagy in M1 macrophages. To determine whether IPA regulates macrophage autophagy via PPT1, we compared autophagic flux between the oe‐PPT1 group and oe‐PPT1 + IPA groups (Figure S10A, Supporting Information). IPA treatment altered autophagic flow in the oe‐PPT1+IPA group compared to oe‐PPT1 alone, as evidenced by the formation of LC3B‐II puncta (Figure S10B, Supporting Information). Moreover, IPA reversed the oe‐PPT1‐induced downregulation of LC3B‐II and Beclin1 while suppressing the upregulation of P62, indicating that IPA counteracts PPT1‐mediated autophagy impairment.

**Figure 9:**
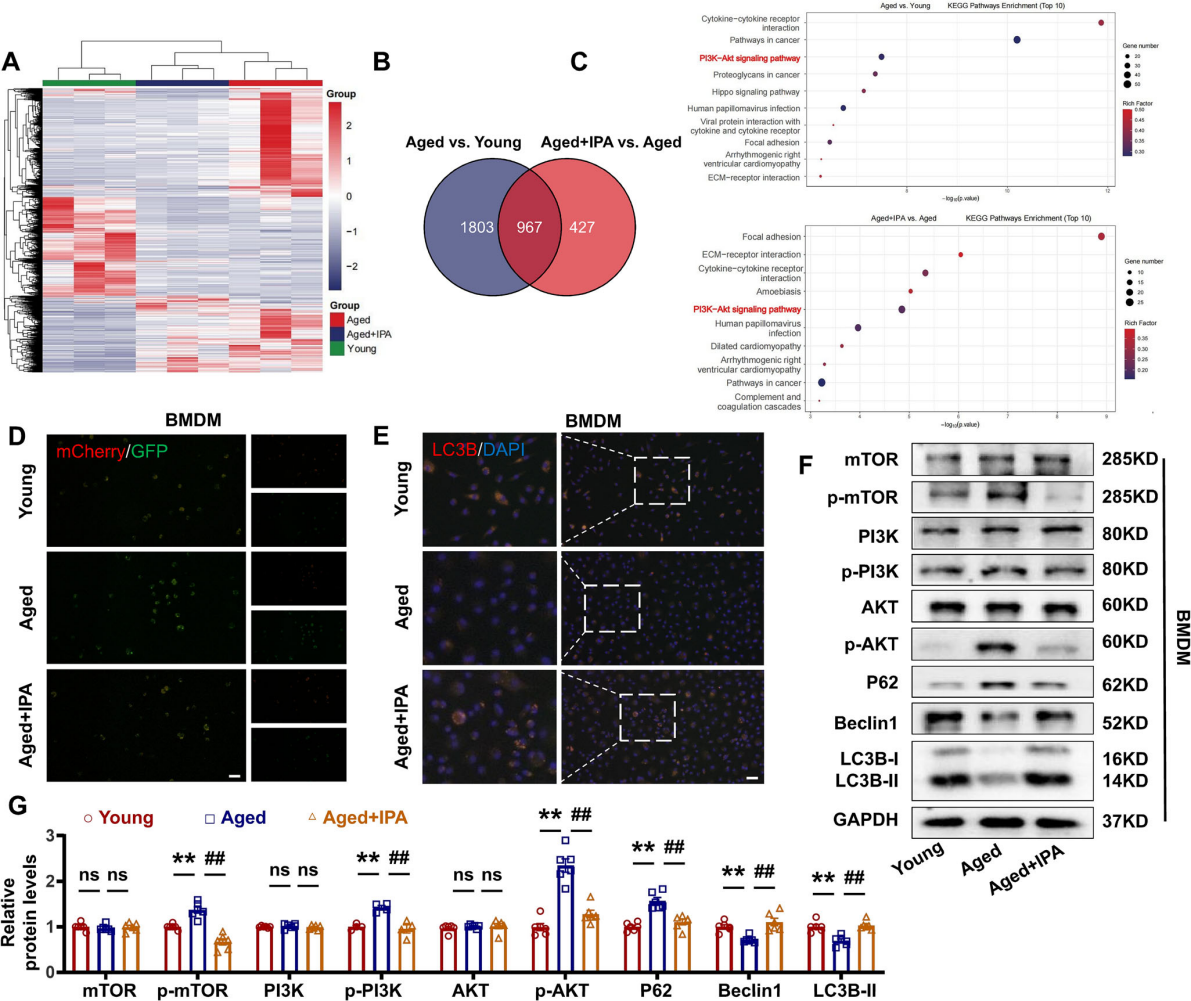
IPA regulates macrophage autophagy via PI3K‐AKT‐mTOR pathway. A) Heat map of differentially expressed genes. B) Venn diagram of differentially expressed genes. C) KEGG pathway enrichment bubble diagram of differentially expressed genes. D) mCherry‐GFP‐LC3B immunofluorescence staining representative images. Magnification: 200×, scale bar = 20 µm. E) Representative images immunofluorescence staining of LC3B. Magnification: 200×, scale bar = 20 µm. F,G) Representative images and statistical analysis of mTOR, p‐mTOR, PI3K, p‐PI3K, AKT, p‐AKT, P62, Beclin1 and LC3B at protein level. (*n* =4–6, data are expressed as mean ± SEM, ^**^ *p <* 0.01 vs the Young group; ^##^ *p <* 0.01 vs the Aged group).

To investigate the mechanism by which IPA enhances lysosomal function and regulates PPT1 activation during aging, we performed RNA‐seq to screen differentially expressed genes. Comparative transcriptomic analysis revealed distinct gene expression profiles among Young‐BMDMs, Aged‐BMDMs, and Aged‐BMDMs treated with IPA (**Figure** **9**A–C). It focused on the KEGG pathway involved in the PI3K‐AKT signaling pathway. To further explore the role of IPA in autophagy regulation, we assessed autophagic flux. Our results demonstrated that IPA treatment increased autophagy levels in aged macrophages (Figure 9D). Fluorescence imaging of LC3B revealed distinct punctate structures in Young and Aged + IPA macrophages, indicating the activation of autophagy (Figure 9E). Western blot analysis further confirmed that IPA modulated the expression of autophagy‐associated marker proteins, including LC3B‐II, Beclin1, and P62, by activating the PI3K‐Akt‐mTOR signaling pathway (Figure 9F,G).

### 2.7 IPA Promotes PPT1 Expression via the cGAS‐STING Pathway

**Figure 10:**
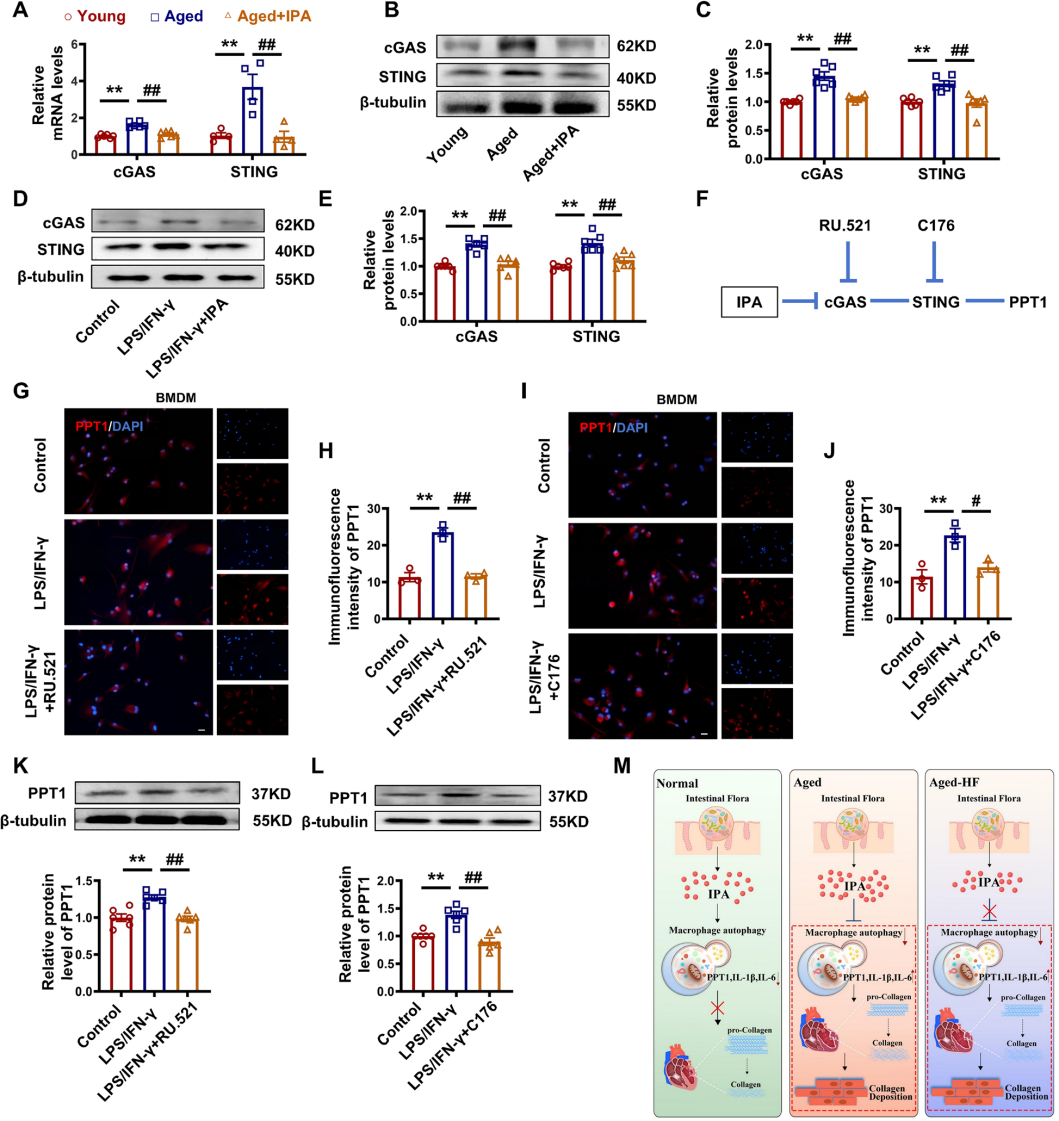
cGAS‐STING pathway is involved in IPA‐induced inhibition of PPT1. A) Statistical graph of cGAS and STING mRNA levels in macrophages. B,C) Representative images and statistical graph of cGAS and STING at protein levels in macrophages. D,E) Representative images and statistical graph of cGAS and STING at protein levels in vitro. F) Schematic diagram of IPA. G–J) Representative images and statistical graph of PPT1 immunofluorescence intensity in M1 macrophages after treated with RU.521 or C176. Magnification: 200×, scale bar = 20 µm. K,L) Representative images and statistical graph of PPT1 protein levels in M1 macrophages after treated with RU.521 or C176. M) Graphical abstract. (*n* = 3–6, data are expressed as mean ± SEM, ^*^ *p <* 0.05, ^**^ *p <* 0.01 vs the Young or Control group; ^#^ *p <* 0.05, ^##^ *p <* 0.01 vs the Aged or LPS/IFN‐γ group).

As an important innate immune signaling pathway, the cGAS‐STING pathway is activated by nucleic acids. It is an important DNA sensor associated with the secretion of pro‐inflammatory factors and is linked to the senescence‐associated secretory phenotype (SASP).^[^ ^29^, ^30^ ^]^ To verify the pathway through which IPA affects the PPT1 protein, we first assessed the impact of IPA on cGAS‐STING expression. Our results showed that IPA treatment reduced the mRNA and protein levels of cGAS and STING in cardiac tissue (**Figure** **10**A–C). These findings were corroborated by in vitro protein analysis (Figure 10D,E). To further validate the role of the cGAS‐STING pathway in PPT1 regulation, we used specific inhibitors: RU.521 (a cGAS inhibitor) and C176 (a STING inhibitor). Inhibition of this pathway led to a significant reduction in PPT1 expression in M1 macrophages (Figure 10F), as confirmed by immunofluorescence and western blotting (Figure 10G–L).

## 3 Discussion

Numerous studies have demonstrated that gut microbiota has been associated with cardiovascular disease, with gut dysbiosis implicated in the development of myocardial fibrosis.^[^ ^31^ ^]^ Previous research highlights the involvement of gut microbiota in inflammatory conditions and emphasizing its broad impact on inflammatory processes.^[^ ^32^, ^33^, ^34^ ^]^ In our study, we found that plasma IPA levels were significantly lower in older patients with heart failure compared to age‐matched individuals without heart disease. Furthermore, our findings highlight the therapeutic potential of IPA, a product of tryptophan metabolism by *c_Clostridium*, in ameliorating cardiac function in aging. Previous studies have shown that pretreatment with IPA attenuates LPS‐induced cardiac dysfunction and inflammation.^[^ ^35^ ^]^ Wang et al. reported that IPA protects against heart failure by preserving ejection fraction.^[^ ^8^ ^]^ Aging induces a wide range of cardiovascular alterations, including extracellular matrix remodeling, increased oxidative stress, and dysregulation of immune responses.^[^ ^36^ ^]^ However, the role of IPA in aging‐related heart disease has not been extensively reported. To address this gap, we found the protective effect of IPA on cardiac function in aged rats, focusing on its ability to reduce inflammation and collagen deposition in aging‐related myocardial fibrosis. Our findings position IPA as a clinically relevant therapeutic drug for cardiovascular health.

Furthermore, we investigated PPT1, a lysosomal enzyme that has been extensively studied in other contexts, including neurodegenerative diseases and systemic autoimmunity, but remains underexplored in the cardiovascular system.^[^ ^18^, ^37^ ^]^ Using proteomic sequencing, this study is the first to identify the key role of PPT1 in the heart. Our research demonstrates that PPT1 expression is upregulated in aged and inflamed cardiac tissue and is mainly regulated by macrophages. scRNA‐seq analysis demonstrated that PPT1 was significantly upregulated in aged macrophages, with PPT1 high expressively macrophages exhibiting a pro‐inflammatory phenotype. These findings suggest that PPT1 may be a potential therapeutic target for treating cardiac inflammation. Long‐term administration of D‐gal in rodents can mimic the overproduction of reactive oxygen species caused by physiological aging, a common way to induce aging in animals in vivo.^[^ ^38^, ^39^ ^]^ By verifying transgenic mice with PPT1 knockout in macrophages, we found that knocking out PPT1 in macrophages could significantly reduce D‐gal‐induced aging‐related myocardial fibrosis and inflammation in mice. Pathological analysis further confirmed that, compared to the D‐gal‐treated group, the D‐gal + cko‐PPT1 group exhibited a significant reduction in cardiac inflammation and collagen deposition. These findings were corroborated by experiments in aged rats, where AAV‐sh‐PPT1 also led to a significant reduction in cardiac fibrosis and inflammation. Inhibition of PPT1 mitigated cardiac fibrosis and alleviated age‐related inflammation, highlighting its potential as a therapeutic target. Aged bone marrow macrophages could drive systemic aging and age‐related dysfunction.^[^ ^40^ ^]^ To determine whether IPA alleviates myocardial fibrosis through macrophages or whether knocking down PPT1 in macrophages can alleviate myocardial fibrosis, we adoptively transferred aged BMDMs into young rats. Our findings revealed that aged macrophages with PPT1 knockdown and aged macrophages treated with IPA significantly reduced inflammation and fibrosis compared to untreated aged macrophages. Additionally, the same conclusion was obtained when the conditioned medium of macrophages was applied to cardiac fibroblasts in vitro. Specifically, COL3A1, COL1A1, and α‐SMA were upregulated in Aged or oe‐PPT1 conditional medium groups, whereas their expression was significantly down‐regulated in aged or oe‐PPT1 macrophage that incubate with si‐PPT1, DC661, and IPA.

Autophagy is essential for maintaining cardiovascular homeostasis and function. Aging is associated with impaired autophagy, leading to the accumulation of metabolic waste and contributing to various pathological conditions. Notably, autophagy acts as a double‐edged sword, both playing a role in and being influenced by cardiac aging.^[^ ^41^, ^42^, ^43^ ^]^ Previous studies have shown that PPT1 regulates autophagy in the context of tumor immunity and skeletal muscle differentiation.^[^ ^44^, ^45^ ^]^ These results suggest that IPA regulates macrophage autophagy via PPT1 in aging model. To further elucidate the signaling pathway through which IPA regulates autophagy in aging macrophages via PPT1, we performed RNA‐seq analysis. Autophagy presents a dual role in cellular physiology. While appropriate levels of autophagy enhance overall cellular function, excessive and insufficient autophagy may disrupt normal physiological processes and contribute to disease progression.^[^ ^46^ ^]^ The results showed that autophagic function in aged BMDMs was impaired, with significant inhibition of the PI3K‐AKT‐mTOR pathway. Notably, IPA treatment upregulated this pathway. To validate these findings, we conducted in vitro experiments to determine whether IPA could restore PI3K‐AKT‐mTOR pathway activity. Our experimental results demonstrated that incubation with IPA enhanced the autophagic function of pro‐inflammatory macrophages within LPS/IFN‐γ or oe‐PPT1 groups. In contrast, our in vivo data showed that IPA reversed aging‐induced autophagy inhibition in BMDMs, as evidenced by autophagy flux analysis and the expression of autophagy marker proteins. Growing evidence suggests that inhibition of the cGAS‐STING signaling pathway can significantly suppress cardiac hypertrophy and inflammatory cell infiltration.^[^ ^47^ ^]^ The cGAS‐STING pathway indirectly promotes aging, primarily through cellular senescence. Our results suggest that the intestinal metabolite IPA reduces cGAS and STING levels in the hearts of aged rats and M1 macrophages. RU.521 or C176 are commonly used inhibitors of cGAS and STING, respectively.^[^ ^48^, ^49^ ^]^ When M1 macrophages were incubated with RU.521 and C176, PPT1 expression decreased, supporting the conclusion that IPA regulates PPT1 expression via the cGAS‐STING pathway. In conclusion, we demonstrated in vivo and in vitro that IPA alleviates aging‐related myocardial fibrosis through PPT1. Additionally, we found that inhibiting PPT1 expression indirectly mitigates aging‐related myocardial fibrosis by modulating the autophagic function of macrophages (Figure 10M).

This study primarily focused on the anti‐inflammatory and antifibrotic functions of IPA but did not investigate the gut microbiota responsible for its production. Future studies will employ fecal microbiota transplantation (FMT) experiments to identify specific gut microbiota influencing IPA levels in older individuals and determine the effect of IPA on the gut microbiota in the elderly. Because natural aging in mice takes ≈16 months, we utilized D‐gal to induce aging. However, we plan to conduct additional experiments using 16‐month‐old cko‐PPT1 mice for further validation. Furthermore, in addition to its role in autophagy, PPT1 functions as a depalmitoylation enzyme, regulating palmitoylation modifications in cardiac disease. We would examine its involvement in the palmitoylation of cardiac tissue of cko‐PPT1 mice in the future. Although the cGAS‐STING pathway plays a pivotal role in autophagy, our study only explored the regulatory relationship between IPA and PPT1. Future research should investigate exact role of this pathway in cardiac aging and myocardial fibrosis.

## 4 Experimental Section

### Clinical Study Subjects

Fourteen healthy young people, 30 healthy elderly people, and 30 elderly patients with obvious symptoms, signs, and diagnosis of heart failure were recruited from the physical examination center who were hospitalized in the Department of Cardiology of the First Affiliated Hospital of Harbin Medical University. All patients underwent routine physical examination, laboratory tests (blood routine, blood biochemistry), electrocardiogram and cardiac color Doppler ultrasound within 24 h after admission. 3 mL peripheral venous blood was collected using EDTA anticoagulated blood collection tubes, and the plasma was separated. The upper layer of plasma was divided and stored at −80 °C. This study was approved by the Ethics Committee of the First Affiliated Hospital of Harbin Medical University.

### Animals Models

All the animal experiments in this study were conducted by the Guide for the Care and Use of Laboratory Animals and approved by the Institutional Animal Care and Use Committee of the First Affiliated Hospital of Harbin Medical University (IACUC No. 2023084). Male SD rats were purchased from Beijing VitalRiver Laboratory Animal Technology Co, Ltd (Beijing, China). Defined 4–6 months old rats were young group and 18–20 months old as aged group. The PPT1 conditional KO mice were generated by employing the CRISPR/Cas9 technology to disrupt macrophage PPT1 and purchased from Cyagen Biosciences (Suzhou) Inc. Animals were raised at the Experimental Animal Center of the First Affiliated Hospital of Harbin Medical University. The animals were individually housed under 12:12‐h light‐dark cycles and were offered water and food ad libitum. At the end of the feeding regimen, the rats were anesthetized with 3% pentobarbital sodium and sacrificed by deep anesthesia of pentobarbital sodium.

### Echocardiographic Measurement

Ehocardiography was performed on each rat after transplantation using echocardiography analysis system. After moderate anesthesia of isoflurane gas in each group of mice, the heart rate was stable. Then, the mice used the short‐axis and long‐axis 2D ultrasound imaging of the heart of the ultrasound system, and analyzed it with the ultrasound imaging analysis system to obtain their heart structure and functional parameters.

### In Vivo PPT1 Knockdown

Knockdown of the PPT1 gene was constructed by Shanghai Genechem Co., Ltd using the recombinant serum type 9 adeno‐associated virus system (AAV9) and the empty vector as the negative control. The GV576‐F4/80p‐MCS‐SV40 PolyA vector contains at least three shRNA fragments that were capable of simultaneously knocking down a gene at multiple sites. These sequences were as follows: 5′‐CGGCATGGACGAGCTGTACAAGTAAGTCTCGAGGGATCCGCTAGC‐3′, 5′‐TTATGATCTAGAGTCGCGGCCGCCAGCTATGGTGGCGGGGCCC‐3′, and 5′‐CCCTGAGCAAAGACCCCAAC‐3′. The virus was injected into 18–20 months old SD rats via the tail vein for 8 weeks.

### Tryptophan Metabolomics

First, the experimental samples were pretreated, the metabolites were extracted, and the raw data were obtained by using the Thermo UltiMate 3000RS and the mass spectrometer Thermo Q Exactive. The raw data were transformed into a matrix for further processing using specialized data processing software, including mass‐to‐charge ratio, retention time, and peak area information of metabolites. Data processing and statistical analysis were performed on the dataset to obtain differential metabolites and perform data visualization analysis. Tryptophan and its metabolites contents were then detected by MetWare Biotechnology Co., Ltd (Wuhan, China).

### Bacteria 16S rDNA Sequencing

Stool samples were collected from young and aged rats, snap‐frozen in liquid nitrogen, and stored at −80 °C. The bacterial 16S rDNA genes in the samples were sequenced by a high‐throughput detection platform. The bacterial genome database was used for data collation and analysis. The data were detected by MetWare Biotechnology Co., Ltd (Wuhan, China).

### Proteomics Sequencing

The isotope labeling quantification technique was used to combine multiple isotope tags with the N‐terminal groups of peptides from multiple groups of samples, and then tandem mass spectrometry was performed. The peak area of the reporter ion was used to calculate the ratio of the same peptide between different samples, so as to realize the quantitative comparison of different samples. The transcriptome sequencing and analysis were conducted by OE Biotech Co., Ltd. (Shanghai, China).

### Single‐Cell and Single‐Nucleus RNA Sequencing Data Processing and Analysis

Single‐cell transcriptomic data (GSE183852) of normal and failing human hearts were downloaded from the Gene Expression Omnibus (GEO) database (https://www.ncbi.nlm.nih.gov/geo/), to characterize age‐associated, macrophage‐specific transcriptional signatures, myeloid cells from healthy donor hearts were selected based on the cell annotations provided in the original publication for downstream analysis. The raw gene expression matrices were first converted into a Seurat object using the Seurat package (v4.3.0) in R. Batch effects were corrected using the Harmony (v1.2.0) algorithm. Cell clustering was conducted with the “FindNeighbors” and “FindClusters” functions in Seurat, using a resolution parameter of 0.5. For visualization, the “RunTSNE” function was used to generate 2D map based on the t‐distributed stochastic neighbor embedding (t‐SNE) algorithm. Clusters expressing marker genes from two or more distinct cell types were considered doublets and excluded from further analysis. Marker genes for each cell population were identified using the “FindAllMarkers” function with default parameters.

### Transcriptome and Bioinformatics Analysis

Total RNA was extracted using the TRIzol reagent (Invitrogen, CA, USA) according to the manufacturer's protocol. RNA purity and quantification were evaluated using the NanoDrop 2000 spectrophotometer (Thermo Scientific, USA). RNA integrity was assessed using the Agilent 2100 Bioanalyzer (Agilent Technologies, Santa Clara, CA, USA). Then the libraries were constructed using VAHTS Universal V6 RNA‐seq Library Prep Kit according to the manufacturer's instructions. The transcriptome sequencing and analysis were conducted by Shanghai Applied Protein Technology Co., Ltd. (Shanghai, China).

### ELISA of IPA

The levels of the IPA in heart tissues were determined using commercial ELISA kits (Jiangsu Meimian Industrial Co., Ltd) according to the manufacturer's instructions.

### Histological Analysis

Histological analysis of was determined with H&E or Masson method. The heart tissues were fixed in 4% paraformaldehyde for 2 days, embedded in paraffin, and sliced into 4µm sections. H&E staining kit (Solarbio, Beijing, China) and Masson's trichrome stain kit (Solarbio, Beijing, China) were used to measure cardiomyocyte morphology and cardiac fibrosis. The myocardial sections were observed by microscope (Carl Zeiss Co., Ltd, Germany).

### Immunohistochemistry Assays (IHC)

Heart sections were deparaffinized in xylene series and rehydrated through ethanol series, followed by treatment of sections with 3% H_2_O_2_ for 10 min. The sections were heated with antigen retrieval buffers to repair the antigen. Then they were blocked with 5% bovine serum albumin at 37 °C for 1 h. Sections were subjected to antigen retrieval in citrate buffer before adding CD68 primary antibody (Santa Cruz, sc‐20060, USA), MCP‐1 primary antibody (Affinity Biosciences, DF7577, China), TNF‐α primary antibody (Proteintech, 66291‐1‐Ig, USA), COL1A1 primary antibody (Proteintech, 66761‐1‐Ig, USA), and α‐SMA (Abcam, ab124964, Great Britain) overnight at 4 °C. The following day, the sections were incubated with horseradish peroxidase (HRP)‐conjugated anti‐mouse antibody or anti‐rabbit antibody and stained with diaminobenzidine (DAB). Images were taken with the microscope (Carl Zeiss Co. Ltd., Germany).

### Primary Culture of Rat Bone Marrow Macrophages

Rats were sacrificed and femur and tibia were separated under sterile conditions and then placed into cell culture dishes containing 75% alcohol. The bone ends of the femur and tibia were cut off, and then bone marrow cells were washed from one of the broken ends of the bone into 10 mL sterile centrifuge tubes using DMEM cell medium and repeated three times. At 1000 r min^−1^, the samples were centrifuged for 8 min and the supernatant was discarded. 5 mL of erythrocyte lysate was added, pipetted repeatedly, and then allowed to rest for 3 min at 1000 r min^−1^, centrifuged for 8 min, and the supernatant was discarded. Cells were resuspended by adding 5 mL DMEM cell medium and filtered through nylon filters. At 1000 r min^−1^, the samples were centrifuged for 8 min, and the supernatant was discarded and repeated three times. Cells were resuspended using a cell culture medium containing DMEM (10% FBS), resuspended in DMEM (10% FBS) containing 20 ng mL^−1^ M‐CSF, and seeded into cell culture dishes. The cells were incubated at 37 °C in a 5% CO_2_ incubator using DMEM medium containing 10 % fetal bovine serum.

### Cell Culture

Primary rat cardiac fibroblasts were cultured as previously described.^[^ ^50^ ^]^ Cardiac fibroblasts (CFs) were acutely isolated from neonatal SD rats (1–3‐day‐old). CFs cells were cultured under standard conditions (37 °C, 5% CO_2_). The cells were divided into different groups and subjected to experimental procedures at 80 % confluence. BMDMs were isolated from Young or Aged rats, cells were treated with 1 µmol L^−1^ IPA or 0.1 µmol L^−1^ DC661 for 24 h.

### Cell Transfection

The pcDNA3.1‐PPT1 plasmid (oe‐PPT1) and pcDNA3.1‐negative control (Vector) were provided by Generalbiol (Chuzhou, China). Small interfering RNA of PPT1 (si‐PPT1) and negative control (NC) were provided by Seven (Beijing, China). Knockdown of the PPT1 gene was constructed by Shanghai Genechem Co., Ltd. by shRNA. When the growth density of BMDMs in six‐well plates reached 80% confluence, transfection was performed using jetPRIME® transfection reagent (Polyplus, France). After 6 h, the medium was replaced with fresh medium, IPA was added to continue the culture for 24 h, and the BMDMs were collected and tested.

### Western Blot Analysis

Cell or tissue samples were lysed in a lysis buffer with protease inhibitors. Subsequently, protein samples were separated by 10% SDS‐PAGE and transferred onto nitrocellulose membranes. The membrane was blocked with 3% no‐fat milk in PBST (PBS with 0.1% Tween 20) at 37 °C for 1 h and incubated with the antibodies overnight at 4 °C, and then with HRP goat anti‐mouse IgG (H + L) or HRP goat anti‐rabbit IgG (H + L) secondary antibody for 1 h at 37 °C. Blot visualization was performed according to the manufacturer's instructions by using a Fully Automatic Chemiluminescence Image Analysis System. The antibody resources are as follows:

ANP primary antibody (Proteintech, 27426‐1‐Ig, USA), β‐tubulin primary antibody (Proteintech, 10094‐1‐Ig, USA), STING primary antibody (Proteintech, 19851‐1‐Ig, USA), AKT primary antibody (Proteintech, 60203‐2‐Ig, USA), PPT1 primary antibody (Proteintech, 29653‐1‐Ig, USA), COL1A1 primary antibody (Proteintech, 66761‐1‐Ig, USA), TNF‐α primary antibody (Proteintech, 60291‐1‐Ig, USA), cGAS primary antibody (ABclonal, A8335, China), PI3K primary antibody (Affinity Biosciences, AF6241, China), p‐PI3K primary antibody (CST, 4228S, USA), p‐AKT primary antibody (Affinity Biosciences, AF0016, China), MCP‐1 primary antibody (Affinity Biosciences, DF7577, China), IL‐6 primary antibody (Bioss, bs‐0782, China), p‐mTOR primary antibody (CST, 5536T, USA), mTOR primary antibody (CST, 2983T, USA), CD68 primary antibody (Santa Cruz, sc‐20060, USA), P16 primary antibody (Santa Cruz, sc‐1661, USA), CD86 primary antibody (Santa Cruz, sc‐28347, USA), α‐SMA primary antibody (Abcam, ab124964, Great Britain), LC3B primary antibody (Abcam, ab192890, Great Britain), P62 primary antibody (Abcam, ab109012, Great Britain), Beclin1 primary antibody (Abcam, ab19662, Great Britain), COL3A1 primary antibody (Abcam, ab184993, Great Britain), iNOS primary antibody (Abcam, ab178945, Great Britain), P21 primary antibody (Novus‐Bio, NBP2‐29463, USA), GAPDH primary antibody (ZSGB‐Bio, TA‐08, China),

### Real‐Time Quantitative Reverse Transcription PCR (RT‐qPCR)

The samples were lysed using the RNAkey Reagent (Seven, Beijing, China), and the concentration and integrity of RNA samples were detected with the Nanodrop 2000 spectrophotometer (Thermo Scientific, USA). The volume of total RNA required for reverse transcription was calculated, and the first‐strand cDNA was synthesized according to the instructions of the FastKing RT Kit (With gDNase). The cDNA was synthesized with 1 µg of total RNA following a Reverse transcription kit (Yeasen, 11141ES60, China). RT‐qPCR was processed by the SYBR Green Mix (Yeasen, 11184ES08, China) and detected the mRNA expression by the SLAN‐96S system (SLAN, China). The data were analyzed using the 2^−ΔΔCT^ method to quantify relative gene expression. The primers are as follows:

Rattus norvegicus:

TNF‐α Forward 5′‐ATCGGTCCCAACAAGGAGGA‐3′,

TNF‐α Reverse: 3′‐CGCTTGGTGGTTTGCTACG‐5′.

PPT1 Forward 5′‐GTACTGGCATGACCCCATCA‐3′,

PPT1 Reverse: 3′‐CAGAGTCTACAGGGTCCACG‐5′.

GAPDH Forward 5′‐CAACTCCCTCAAGATTGTCAGCAA‐3′,

GAPDH Reverse: 3′‐GGCATGGACTGTGGTCATGA‐5′.

IL‐6 Forward 5′‐CCAGTTGCCTTCTTGGGACT‐3′,

IL‐6 Reverse: 3′‐CTGGTCTGTTGTGGGTGGTA‐5′.

COL1A1 Forward 5′‐GAGACAGGCGAACAAGGTGA‐3′,

COL1A1 Reverse: 3′‐GGGAGACCGTTGAGTCCATC‐5′.

α‐SMA Forward 5′‐ACCATCGGGAATGAACGCTT‐3′,

α‐SMA Reverse: 3′‐CTGTCAGCAATGCCTGGGTA‐5′.

P21 Forward 5′‐GCACTCTGAAGATGTGCCTATG‐3′,

P21 Reverse: 3′‐CCCATAACTCCTTGCTAACTCC‐5′.

COL3A1 Forward 5′‐CAGTCATGGGACTGGCATTTA‐3′,

COL3A1 Reverse: 3′‐CAGTCATGGGACTGGCATTTA‐5′.

cGAS Forward 5′‐GAAGCCCTGCTGTAACCCTT‐3′,

cGAS Reverse: 3′‐ACAAGATAAAACGGCTCTCGTC‐5′.

STING Forward 5′‐CCCAGCAAAACACAGACCG‐3′,

STING Reverse: 3′‐AAACAAGGTCTGCAAGGGGGT‐5′.

Mus musculus:

PPT1 Forward 5′‐GTCTTTGGACTCCCCCGATG‐3′,

PPT1 Reverse: 3′–CAGGCGTTCTTGCACAAGTT5′.

GAPDH Forward 5′‐TGTGTCCGTCGTGGATCTGA‐3′,

GAPDH Reverse: 3′‐TTGCTGTTGAAGTCGCAGGAG‐5′.

IL‐6 Forward 5′‐TTCCTCTGGTCTTCTGGAGT‐3′,

IL‐6 Reverse: 3′‐TCTGTGACTCCAGCTTATCTCTTG‐5′.

COL1A1 Forward 5′‐AGACCTGTGTGTTCCCTACT‐3′,

COL1A1 Reverse: 3′‐GAATCCATCGGTCATGCTCTC‐5′.

COL3A1 Forward 5′‐ACGTAAGCACTGGTGGACAG‐3′,

COL3A1 Reverse: 3′‐CAGGAGGGCCATAGCTGAAC‐5′.

### Immunofluorescence Assay

Wash samples two times with PBS. Fix samples with 4% paraformaldehyde in PBS for 15 min at room temperature. Permeabilize samples with 0.1% triton x‐100 in PBS for 10 min. Incubate samples in 10% normal goat serum in PBS for 30 min at room temperature. Samples were stained with primary antibody for 16 h. Then, the secondary antibody Alexa Fluor 488 or 647‐labeled Goat Anti‐Rabbit or Mouse IgG(H+L), was added for 1 h. DAPI was added for 5 min. Immunofluorescence was detected by microscope (Carl Zeiss, Germany).

### Statistical Analysis

Statistical analyses were performed using GraphPad Prism 9.0 software (GraphPad Software, Inc., La Jolla, CA, USA). Continuous variables are presented as the mean ± standard error of the mean (SEM), while categorical variables are expressed as numbers and percentages. Two‐group comparisons were performed using the Student's unpaired *t*‐test. For comparisons among multiple groups, a one‐way analysis of variance (ANOVA) was used. Differences were considered statistically significant at ^*^ *p <* 0.05, ^**^ *p <* 0.01.

## Conflict of Interest

The authors declare no conflict of interest.

## Author Contributions

J.L., H.Y.W., and H.Y.Z. contributed equally to this work. J.L., Z.X.D., Y.L., and Y.Q.S. designing research studies. J.L., H.Y.W., J.H.L., and Q.Y.C. conducting experiments. H.Y.Z. and J.X. acquiring data. H.Y.W., H.Y.Z., J.H.L., and J.X. analyzing data. H.Q.L., D.Y.H., J.J.L., L.L., K.Y.Y., X.P.W., S.Q.S., and F.K.L. providing reagents. J.L. and Z.X.D. writing the manuscript.

## Supporting information



Supporting Information

## Data Availability

The data that support the findings of this study are available from the corresponding author upon reasonable request.

## References

[1] C. Zhang , B. Xie , X. Wang , M. Pan , J. Wang , H. Ding , T. Li , H. Lin , Z. Gu , Public Health 2024, 230, 66. 38507918 10.1016/j.puhe.2024.02.015

[2] Y. Luo , J. Lu , Z. Wang , L. Wang , G. Wu , Y. Guo , Z. Dong , Pharm. Biol. 2022, 60, 1762. 36086802 10.1080/13880209.2022.2101672 PMC9467557

[3] A. Tamiato , L. S. Tombor , A. Fischer , M. Muhly‐Reinholz , L. R. Vanicek , B. N. Toğru , J. Neitz , S. F. Glaser , M. Merten , D. R. Morales , J. Kwon , S. Klatt , B. Schumacher , S. Günther , W. T. Abplanalp , D. John , I. Fleming , N. Wettschureck , S. Dimmeler , G. Luxán , Circ. Res. 2024, 134, 1240. 38563133 10.1161/CIRCRESAHA.123.324183 PMC11081481

[4] Y. Li , Y. Liu , J. Cui , M. Zhu , W. Wang , K. Chen , L. Huang , Y. Liu , Cardiovasc. Diabetol. 2024, 23, 123. 38581039 10.1186/s12933-024-02217-y PMC10998415

[5] E. N. DeJong , M. G. Surette , D. M. E. Bowdish , Cell Host Microbe 2020, 28, 180. 32791111 10.1016/j.chom.2020.07.013

[6] R. Peng , C. Song , S. Gou , H. Liu , H. Kang , Y. Dong , Y. Xu , P. Hu , K. Cai , Q. Feng , H. Guan , F. Li , Pharmacol. Res. 2024, 202, 107121. 38431091 10.1016/j.phrs.2024.107121

[7] M. Gesper , A. B. H. Nonnast , N. Kumowski , R. Stoehr , K. Schuett , N. Marx , B. A. Kappel , Front. Med. (Lausanne) 2021, 8, 648259. 33829028 10.3389/fmed.2021.648259 PMC8019752

[8] Y.‐C. Wang , Y. C. Koay , C. Pan , Z. Zhou , W. Tang , J. Wilcox , X. S. Li , A. Zagouras , F. Marques , H. Allayee , F. E. Rey , D. M. Kaye , J. F. O'Sullivan , S. L. Hazen , Y. Cao , A. J. Lusis , Circ. Res. 2024, 134, 371. 38264909 10.1161/CIRCRESAHA.123.322381 PMC10923103

[9] N. S. Esfahani , Q. Wu , N. Kumar , L. P. Ganesan , W. P. Lafuse , M. V. S. Rajaram , Aging Cell 2021, 20, 13438. 10.1111/acel.13438 PMC8373275 34342127

[10] Y. Ma , A. J. Mouton , M. L. Lindsey , Transl. Res. 2018, 191, 15. 29106912 10.1016/j.trsl.2017.10.001 PMC5846093

[11] A. Salminen , K. Kaarniranta , A. Kauppinen , Cell. Mol. Life Sci. 2019, 76, 1901. 30788516 10.1007/s00018-019-03048-x PMC6478639

[12] K. Molawi , Y. Wolf , P. K. Kandalla , J. Favret , N. Hagemeyer , K. Frenzel , A. R. Pinto , K. Klapproth , S. Henri , B. Malissen , H.‐R. Rodewald , N. A. Rosenthal , M. Bajenoff , M. Prinz , S. Jung , M. H. Sieweke , J. Exp. Med. 2014, 211, 2151. 25245760 10.1084/jem.20140639 PMC4203946

[13] Y. A. Chiao , Q. Dai , J. Zhang , J. Lin , E. F. Lopez , S. S. Ahuja , Y.‐M. Chou , M. L. Lindsey , Y.‐F. Jin , Circ. Cardiovasc. Genet. 2011, 4, 455. 21685172 10.1161/CIRCGENETICS.111.959981 PMC3158732

[14] S. Zhang , R. Chen , S. Chakrabarti , Z. Su , Clin. Transl. Immunol. 2020, 9, 1167. 10.1002/cti2.1167 PMC7450172 32874584

[15] J.‐Y. Lu , S. L. Hofmann , J. Inherit. Metab. Dis. 2006, 29, 119. 16601878 10.1007/s10545-006-0225-z

[16] V. W. Rebecca , M. C. Nicastri , N. McLaughlin , C. Fennelly , Q. McAfee , A. Ronghe , M. Nofal , C.‐Y. Lim , E. Witze , C. I. Chude , G. Zhang , G. M. Alicea , S. Piao , S. Murugan , R. Ojha , S. M. Levi , Z. Wei , J. S. Barber‐Rotenberg , M. E. Murphy , G. B. Mills , Y. Lu , J. Rabinowitz , R. Marmorstein , Q. Liu , S. Liu , X. Xu , M. Herlyn , R. Zoncu , D. C. Brady , D. W. Speicher , et al., Cancer Discovery 2017, 7, 1266. 28899863 10.1158/2159-8290.CD-17-0741 PMC5833978

[17] D. Lv , X. Cao , L. Zhong , Y. Dong , Z. Xu , Y. Rong , H. Xu , Z. Wang , H. Yang , R. Yin , M. Chen , C. Ke , Z. Hu , W. Deng , B. Tang , Cell Rep. Med. 2023, 4, 101129. 37480849 10.1016/j.xcrm.2023.101129 PMC10439185

[18] H. Ni , Y. Wang , K. Yao , L. Wang , J. Huang , Y. Xiao , H. Chen , B. Liu , C. Y. Yang , J. Zhao , Nat. Commun. 2024, 15, 1. 38169466 10.1038/s41467-023-43650-z PMC10762000

[19] C. Settembre , R. De Cegli , G. Mansueto , P. K. Saha , F. Vetrini , O. Visvikis , T. Huynh , A. Carissimo , D. Palmer , T. J. Klisch , A. C. Wollenberg , D. Di Bernardo , L. Chan , J. E. Irazoqui , A. Ballabio , Nat. Cell Biol. 2013, 15, 647. 23604321 10.1038/ncb2718 PMC3699877

[20] E. S. Ali , K. Mitra , S. Akter , S. Ramproshad , B. Mondal , I. N. Khan , M. T. Islam , J. Sharifi‐Rad , D. Calina , W. C. Cho , Cancer Cell Int. 2022, 22, 284. 36109789 10.1186/s12935-022-02706-8 PMC9476305

[21] P. Y. Lee , D. B. Sykes , S. Ameri , D. Kalaitzidis , J. F. Charles , N. Nelson‐Maney , K. Wei , P. Cunin , A. Morris , A. E. Cardona , D. E. Root , D. T. Scadden , P. A. Nigrovic , Sci. Immunol. 2017, 2, aam6641. 10.1126/sciimmunol.aam6641 PMC6953719 28763796

[22] Y. Xu , Y. Xiong , Front. Immunol. 2024, 15, 1482738. 39450170 10.3389/fimmu.2024.1482738 PMC11500076

[23] Y. Zhang , W. Chen , Y. Wang , Biomed. Pharmacother. 2020, 125, 110022. 32106379 10.1016/j.biopha.2020.110022

[24] A. K. Sinha , M. F. Laursen , J. E. Brinck , M. L. Rybtke , A. P. Hjørne , N. Procházková , M. Pedersen , H. M. Roager , T. R. Licht , Nat. Microbiol. 2024, 9, 1964. 38918470 10.1038/s41564-024-01737-3 PMC11306097

[25] Z. Wang , Z. Wu , H. Wang , R. Feng , G. Wang , M. Li , S.‐Y. Wang , X. Chen , Y. Su , J. Wang , W. Zhang , Y. Bao , Z. Lan , Z. Song , Y. Wang , X. Luo , L. Zhao , A. Hou , S. Tian , H. Gao , W. Miao , Y. Liu , H. Wang , C. Yin , Z.‐L. Ji , M. Feng , H. Liu , L. Diao , I. Amit , Y. Chen , et al., Cell 2023, 186, 4454. 37703875 10.1016/j.cell.2023.08.019

[26] A. L. Koenig , I. Shchukina , J. Amrute , P. S. Andhey , K. Zaitsev , L. Lai , G. Bajpai , A. Bredemeyer , G. Smith , C. Jones , E. Terrebonne , S. L. Rentschler , M. N. Artyomov , K. J. Lavine , Nat. Cardiovasc. Res. 2022, 1, 263. 35959412 10.1038/s44161-022-00028-6 PMC9364913

[27] M. DeBerge , R. Chaudhary , S. Schroth , E. B. Thorp , JACC Basic Transl. Sci. 2023, 8, 884. 37547069 10.1016/j.jacbts.2022.12.010 PMC10401297

[28] W. Chen , W. Xiao , X. Liu , P. Yuan , S. Zhang , Y. Wang , W. Wu , Bioact. Mater. 2022, 11, 283. 34977432 10.1016/j.bioactmat.2021.09.027 PMC8668428

[29] L. Ou , A. Zhang , Y. Cheng , Y. Chen , Front. Immunol. 2021, 12, 795048. 34956229 10.3389/fimmu.2021.795048 PMC8695770

[30] Y. Wu , Q. Wei , J. Yu , Clin. Interv. Aging 2019, 14, 1277. 31371933 10.2147/CIA.S200637 PMC6628971

[31] H. Xu , F. Yang , Z. Bao , Eur. J. Pharmacol. 2023, 940, 175355. 36309048 10.1016/j.ejphar.2022.175355

[32] J. Li , L. Zhang , T. Wu , Y. Li , X. Zhou , Z. Ruan , J. Agric. Food Chem. 2021, 69, 1487. 33356219 10.1021/acs.jafc.0c05205

[33] Z.‐M. Jiang , S.‐L. Zeng , T.‐Q. Huang , Y. Lin , F.‐F. Wang , X.‐J. Gao , J. Li , P. Li , E.‐H. Liu , Sci. Bull. 2023, 68, 1540. 10.1016/j.scib.2023.06.027 37422372

[34] H. Fang , Y. Wang , J. Deng , H. Zhang , Q. Wu , L. He , J. Xu , X. Shao , X. Ouyang , Z. He , Q. Zhou , H. Wang , Y. Deng , C. Chen , mSystems 2022, 7, 0139921. 10.1128/msystems.01399-21 PMC9239149 35642838

[35] Y. Zhang , S. Li , X. Fan , Y. Wu , J. Inflamm. Res. 2024, 17, 5293. 39157586 10.2147/JIR.S466777 PMC11330251

[36] J. A. Sumner , S. Cleveland , T. Chen , J. L. Gradus , Transl. Psychiatry 2023, 13, 25. 36707505 10.1038/s41398-023-02330-8 PMC9883529

[37] J. Vesa , E. Hellsten , L. A. Verkruyse , L. A. Camp , J. Rapola , P. Santavuori , S. L. Hofmann , L. Peltonen , Nature 1995, 376, 584. 7637805 10.1038/376584a0

[38] L. Zhao , P. Tang , Y. Lin , M. Du , H. Li , L. Jiang , H. Xu , H. Sun , J. Han , Z. Sun , R. Xu , H. Lou , Z. Chen , P. Kopylov , X. Liu , Y. Zhang , Aging Cell 2024, 23, 14063. 10.1111/acel.14063 PMC10928583 38098220

[39] Y. Wang , Y. Xu , W. Guo , Y. Fang , L. Hu , R. Wang , R. Zhao , D. Guo , B. Qi , G. Ren , J. Ren , Y. Li , M. Zhang , Redox Biol. 2022, 58, 102537. 36436456 10.1016/j.redox.2022.102537 PMC9709154

[40] J. Hou , K.‐X. Chen , C. He , X.‐X. Li , M. Huang , Y.‐Z. Jiang , Y.‐R. Jiao , Q.‐N. Xiao , W.‐Z. He , L. Liu , N.‐Y. Zou , M. Huang , J. Wei , Y. Xiao , M. Yang , X.‐H. Luo , C. Zeng , G.‐H. Lei , C.‐J. Li , Nat Aging 2024, 4, 1562. 39266768 10.1038/s43587-024-00694-0 PMC11564114

[41] M. Abdellatif , L. Montégut , G. Kroemer , Eur. Heart J. 2023, 44, 4819. 37832515 10.1093/eurheartj/ehad661

[42] J. Mialet‐Perez , C. Vindis , Essays Biochem. 2017, 61, 721. 29233881 10.1042/EBC20170022

[43] G. Műzes , F. Sipos , Biomedicines 2023, 11, 1130. 36672697 10.3390/biomedicines11010189 PMC9855358

[44] H. R. Yun , Y. H. Jo , J. Kim , N. N. Y. Nguyen , Y. Shin , S. S. Kim , T. G. Choi , Front. Physiol. 2020, 11, 569221. 33178040 10.3389/fphys.2020.569221 PMC7593845

[45] M. Bhardwaj , J. J. Lee , A. M. Versace , S. L. Harper , A. R. Goldman , M. A. S. Crissey , V. Jain , M. P. Singh , M. Vernon , A. E. Aplin , S. Lee , M. Morita , J. D. Winkler , Q. Liu , D. W. Speicher , R. K. Amaravadi , J. Clin. Invest. 2023, 133, 164596. 10.1172/JCI164596 PMC10104903 36795483

[46] S. Sridhar , Y. Botbol , F. Macian , A. M. Cuervo , J. Pathol. 2012, 226, 255. 21990109 10.1002/path.3025 PMC3996449

[47] C. An , Z. Li , Y. Chen , S. Huang , F. Yang , Y. Hu , T. Xu , C. Zhang , S. Ge , Cell Biosci. 2024, 14, 58. 38720328 10.1186/s13578-024-01242-4 PMC11080250

[48] C. Wiser , B. Kim , J. Vincent , M. Ascano , Sci. Rep. 2020, 10, 7604. 32371942 10.1038/s41598-020-64348-y PMC7200739

[49] P. T. Pham , O. Bavuu , J.‐R. Kim‐Kaneyama , X.‐F. Lei , T. Yamamoto , K. Otsuka , K. Suto , K. Kusunose , S. Yagi , H. Yamada , T. Soeki , M. Shimabukuro , G. N. Barber , M. Sata , D. Fukuda , J. Am. Heart Assoc. 2023, 12, 030084. 10.1161/JAHA.123.030084 PMC10727293 37947148

[50] M. Su , J. Wang , C. Wang , X. Wang , W. Dong , W. Qiu , Y. Wang , X. Zhao , Y. Zou , L. Song , L. Zhang , R. Hui , Cell Death Differ. 2015, 22, 986. 25394488 10.1038/cdd.2014.187 PMC4423182

