# Supplementary material for: Gut Metabolite Indole‐3‐Propionic Acid Regulates Macrophage Autophagy Through PPT1 Inhibiting Aging‐Related Myocardial Fibrosis
Source: Adv Sci (Weinh). 2025 Jun 20;12(34):e01070. doi: 10.1002/advs.202501070 (PMC12442707; doi:10.1002/advs.202501070)
Supplement: Supplementary file 1 — Supporting Information [file ADVS-12-e01070-s001.docx]

**Gut metabolite indole-3-propionic acid regulates macrophage autophagy through PPT1 inhibiting aging-related myocardial fibrosis**

Jing Lu *et. al.*

*Corresponding author Email:

dongzx@hrbmu.edu.cn.

Yue Li, ly99ly@hrbmu.edu.cn.

Yuanqi Shi, kean1943@hrbmu.edu.cn.

**This file includes:**

Supplementary Figure 1 to 10

Supplementary Table 1


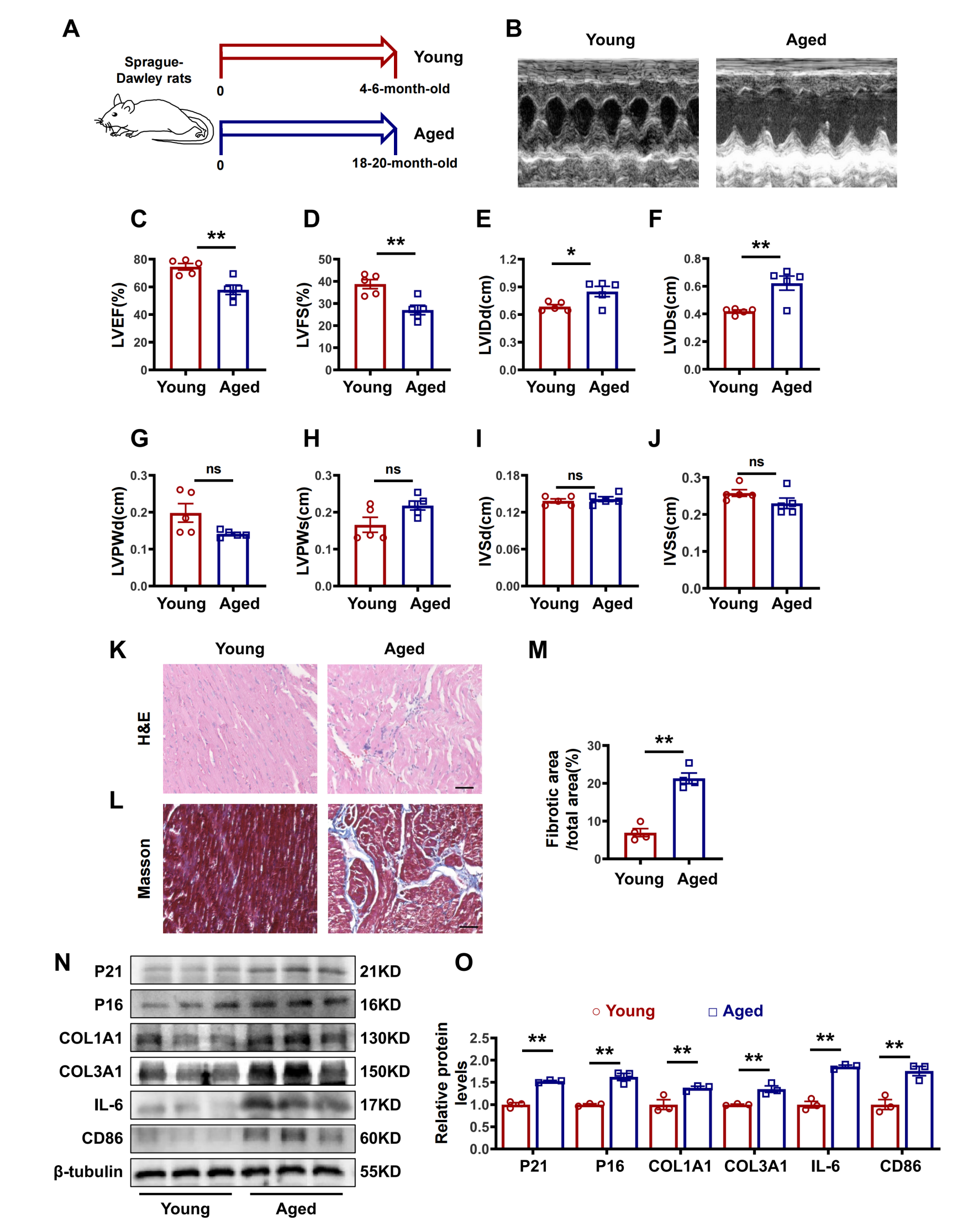


Supplementary Figure 1. Cardiac hypofunction and increased inflammation in aged rats.

**A)** Schematic of the experimental design. **B)** Representative echocardiographic graphs. **C-J)** Echocardiographic measurements of LVEF, LVFS, LVIDd, LVIDs, LVPWd, LVPWs, IVSd and IVSs. **K)** H&E staining of left ventricles. Magnification: 200×, scale bar=50 μm. **L)** Masson’s staining of left ventricles. Magnification: 200×, scale bar=50 μm. **M)** Statistical graphs of Masson’s staining. **N**–**O)** Representative and statistical graphs of western blot of P21, P16, COL1A1, COL3A1, IL-6 and CD86. (n = 3–5, data are expressed as mean ± SEM, ^*^*p* < 0.05, ^**^*p* < 0.01 vs. the Young group).


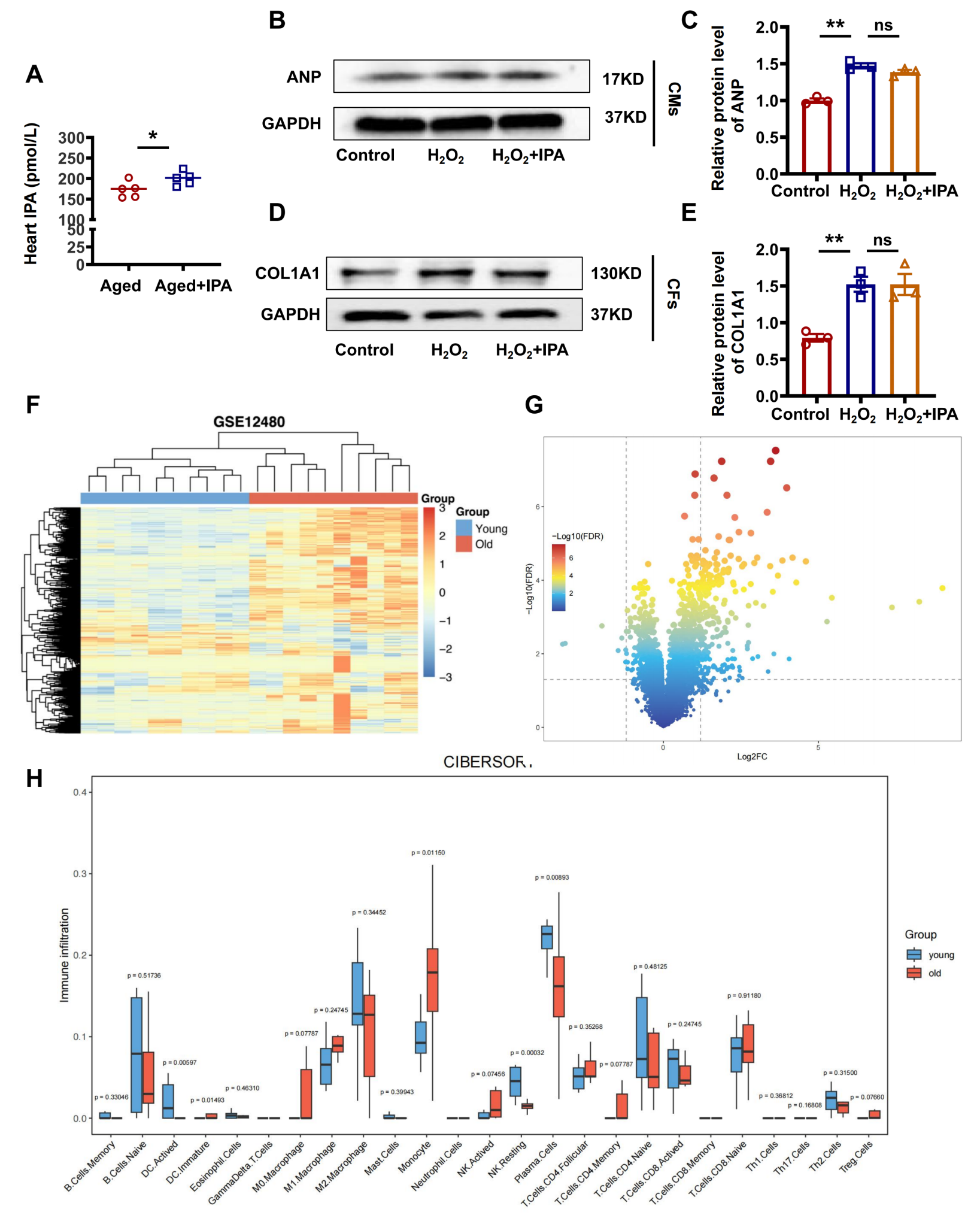


Supplementary Figure 2. Macrophage infiltration in the heart during aging.

A) Heart IPA concentration. B–C) Representative images and statistical graphs of ANP at protein levels in CMs. D–E) Representative images and statistical graphs of COL1A1 at protein levels in CFs. F) Heatmap between Young and Old groups in GEO database: GSE12480. G) Volcano plot of differentially expressed gene. H) CIBERSORT immune infiltration analysis. (n = 3-5, data are expressed as mean ± SEM, ^**^*p* < 0.01 vs. the Control/ Aged group)


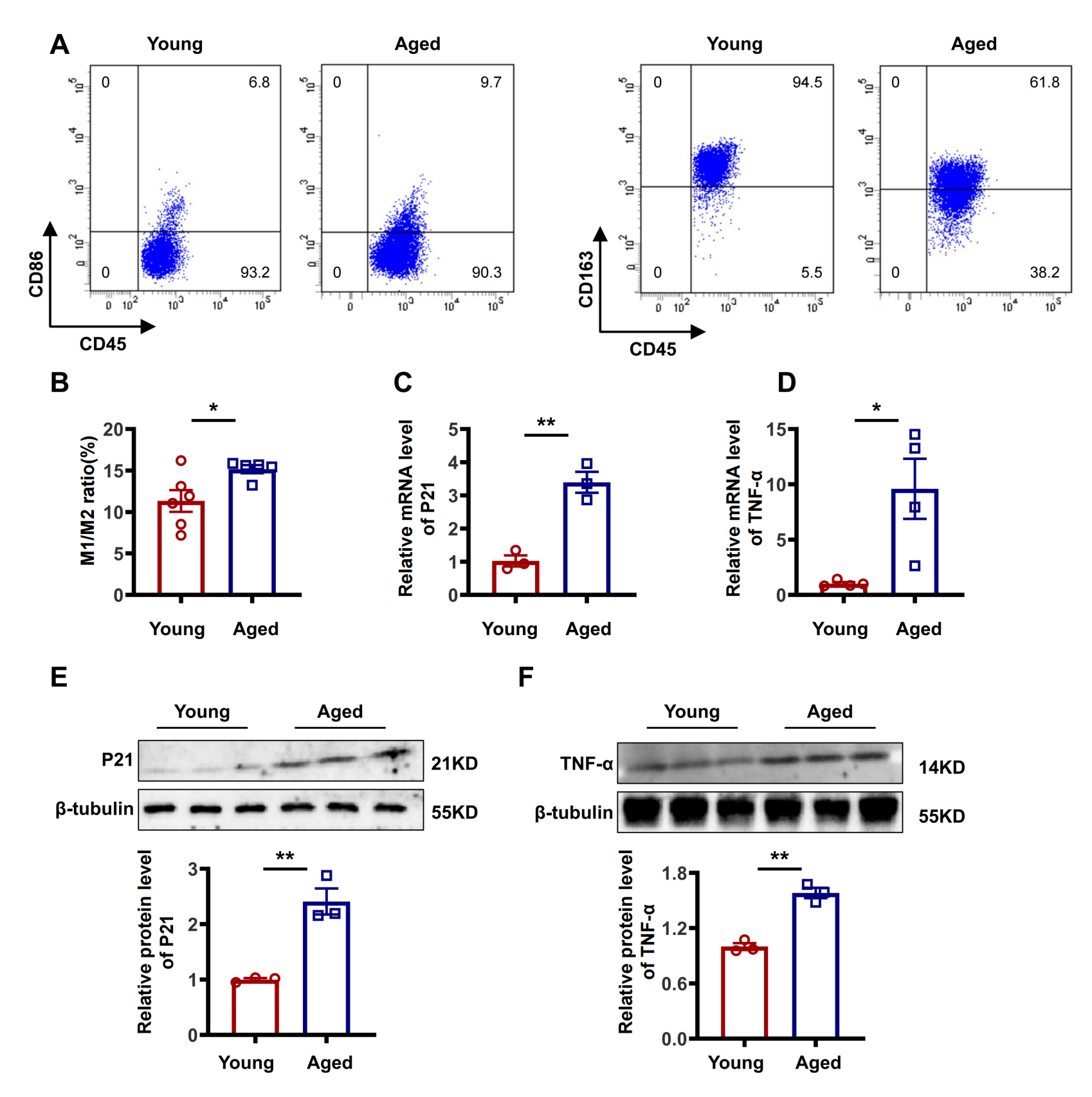


Supplementary Figure 3. Phenotypic verification of senescent macrophages.

**A)** Representative flow cytometry images. **B)** Flow cytometry statistics. M1/M2 ratio=CD45^+^CD86^+^/CD45^+^CD163^+^. **C**–**D)** Statistical graphs of q-PCR of P21 and TNF-α*.* **E**–**F)** Representative images and statistical graphs of P21 and TNF-α at protein levels. (n = 3–6, data are expressed as mean ± SEM, ^*^*p* < 0.05, ^**^*p* < 0.01 vs. the Young group)


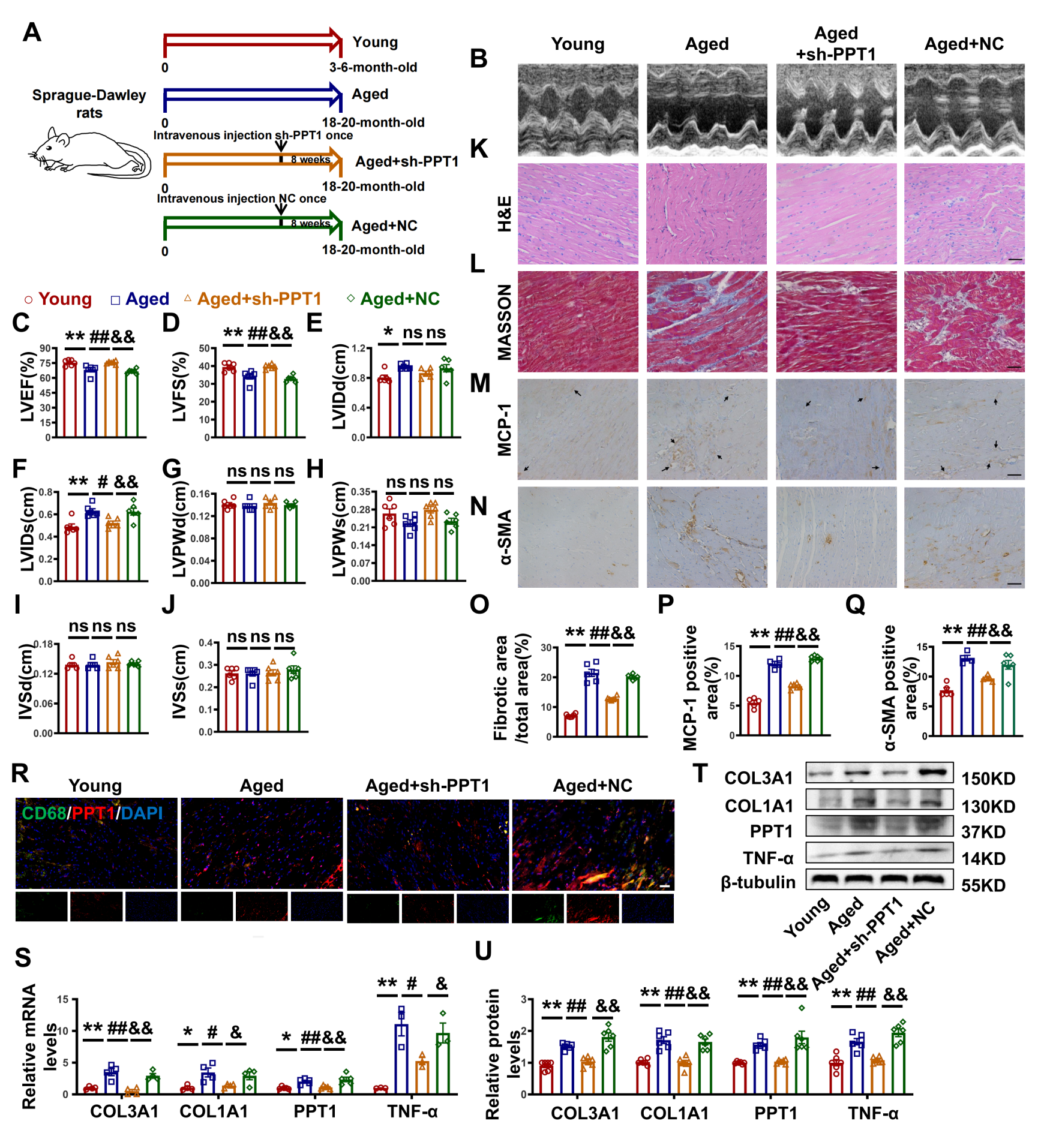


Supplementary Figure 4. Inhibition of macrophage PPT1 improves cardiac inflammatory infiltration and myocardial fibrosis in aged rats

**A)** Schematic of the experimental design. **B)** Representative echocardiographic graphs. **C**–**J)** Echocardiographic measurements of LVEF, LVFS, LVIDd, LVIDs, LVPWd, LVPWs, IVSd and IVSs. **K)** H&E staining of left ventricles. Magnification: 200×, scale bar=50 μm. **L)** Masson’s staining of left ventricles. Magnification: 200×, scale bar=50 μm. **M**–**N)** Representative images of IHC staining with MCP-1 and α-SMA antibody. Magnification: 200×, scale bar=50 μm. **O)** Statistical graphs of Masson’s staining. **P**–**Q)** Statistical graphs of IHC staining with MCP-1 and α-SMA antibody. **R)** Representative images of the immunofluorescence of CD68, PPT1, and DAPI in rat heart. Magnification: 200×, scale bar=20 μm. **S)** Statistical analysis of COL3A1, COL1A1, PPT1 and TNF-α at mRNA level. **T**–**U)** Representative images and statistical analysis of COL3A1, COL1A1, PPT1 and TNF-α at protein level. (n = 3–6, data are expressed as mean ± SEM, ^*^*p* < 0.05, ^**^*p* < 0.01 vs. the Young group; ^#^*p* < 0.05, ^##^*p* < 0.01 vs. the Aged group; ^&^*p* < 0.05, ^&&^*p* < 0.01 vs. the Aged+sh-PPT1 group)


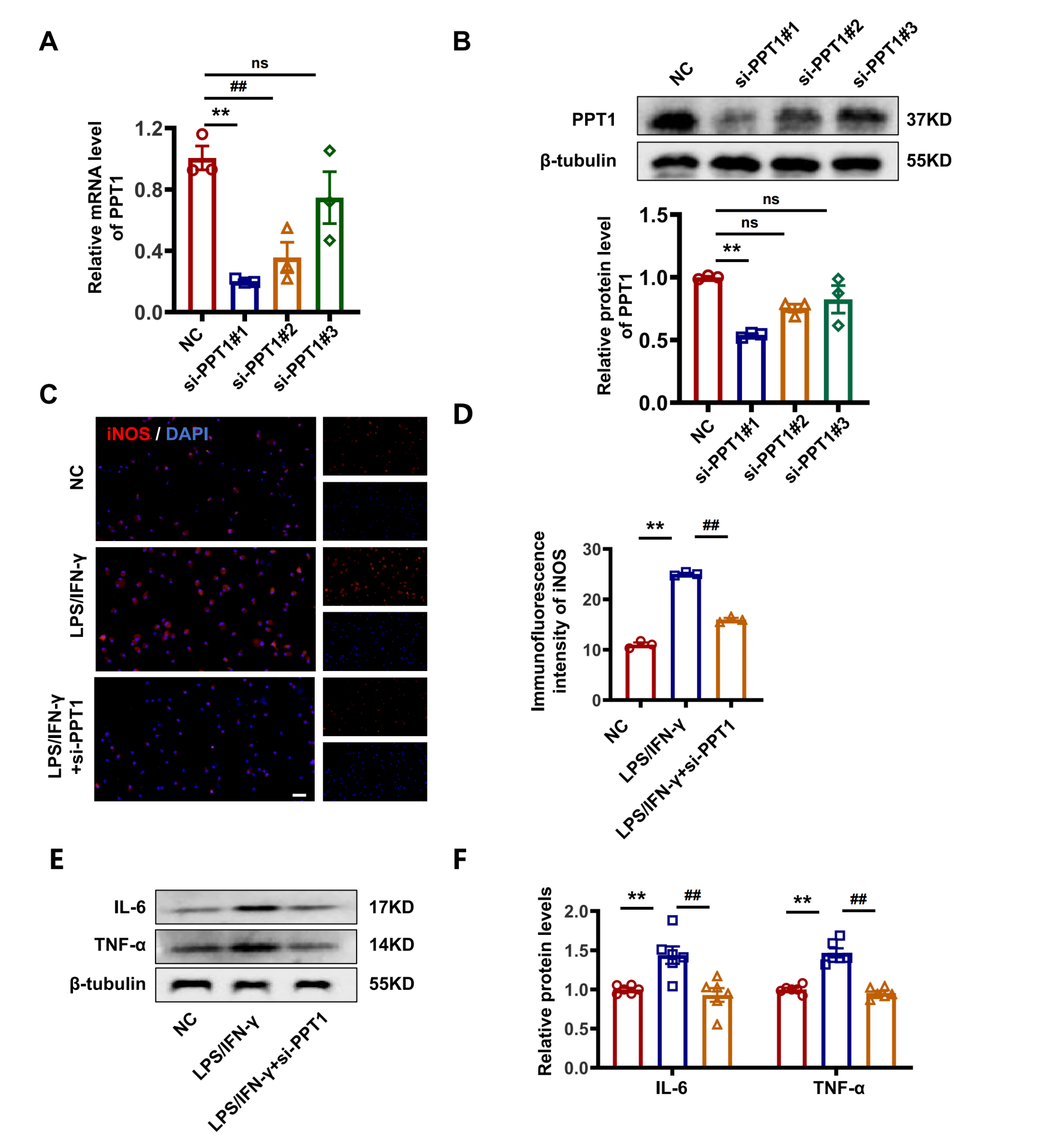


Supplementary Figure 5. Knockdown of PPT1 reduces the secretion of inflammatory factors in macrophages.

**A)** Statistical graph of PPT1 mRNA knockdown efficiency verification. **B)** Representative graphs and statistical graphs for validation of PPT1 protein knockdown efficiency. **C**–**D)** Representative images and statistical graphs of the immunofluorescence of iNOS. Magnification: 200×, scale bar=20 μm. **E**–**F)** Representative images and statistical analysis of IL-6 and TNF-α at protein level. (n = 3–6, data are expressed as mean ± SEM, ^**^*p* < 0.01 vs. the NC group; ^##^*p* < 0.01 vs. NC or LPS/IFN-γ group)


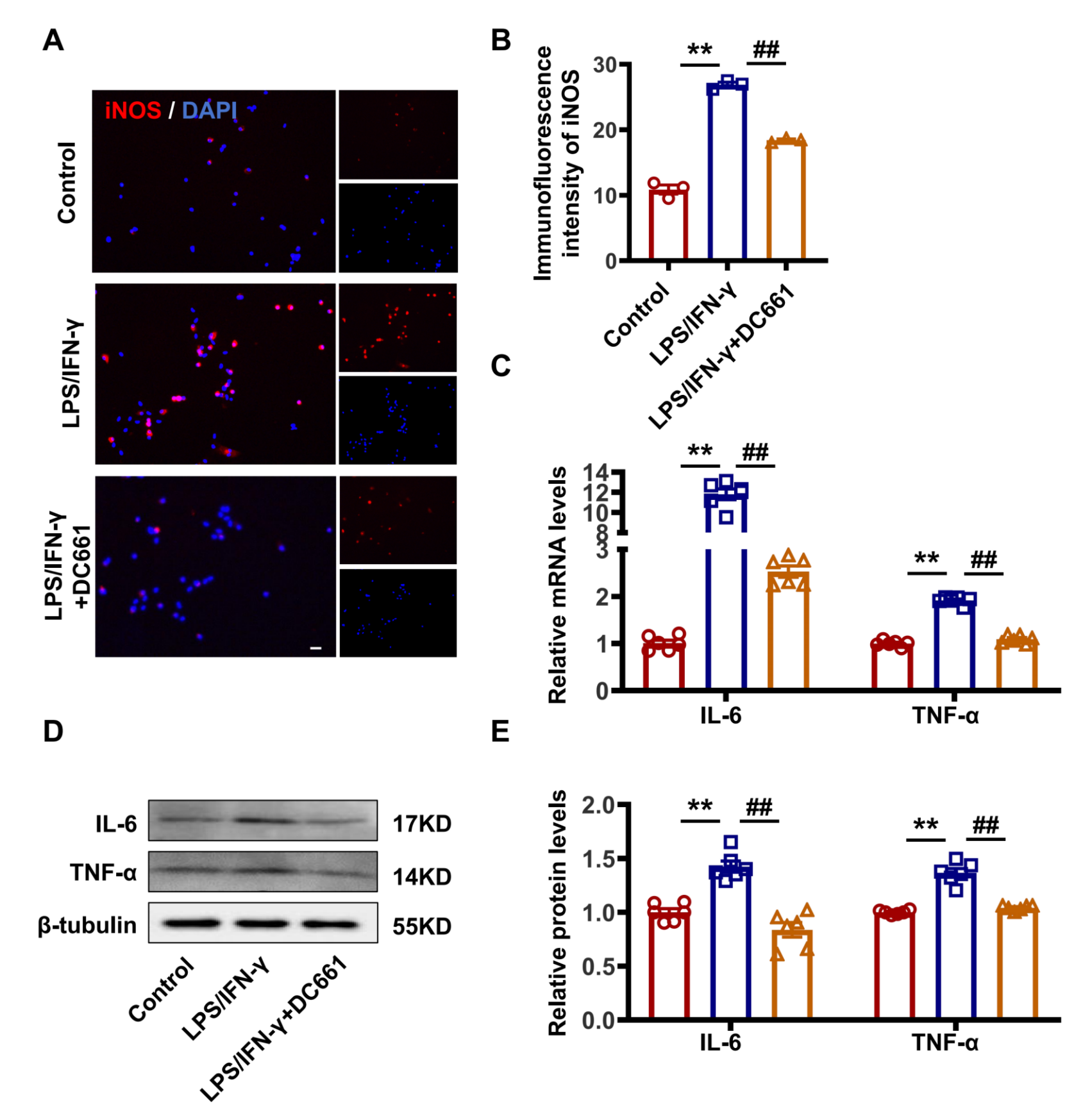


Supplementary Figure 6. PPT1 inhibitor DC661 reduces the secretion of inflammatory factors in macrophages.

**A**–**B)** Representative images and statistical graphs of the immunofluorescence of iNOS. Magnification: 200×, scale bar=20 μm. **C)** Statistical analysis of IL-6 and TNF-α at mRNA level. **D**–**E)** Representative images and statistical analysis of IL-6 and TNF-α at protein level. (n = 3–6, data are expressed as mean ± SEM, ^**^*p* < 0.01 vs. the Control group; ^##^*p* < 0.01 vs. the LPS/IFN-γ group)


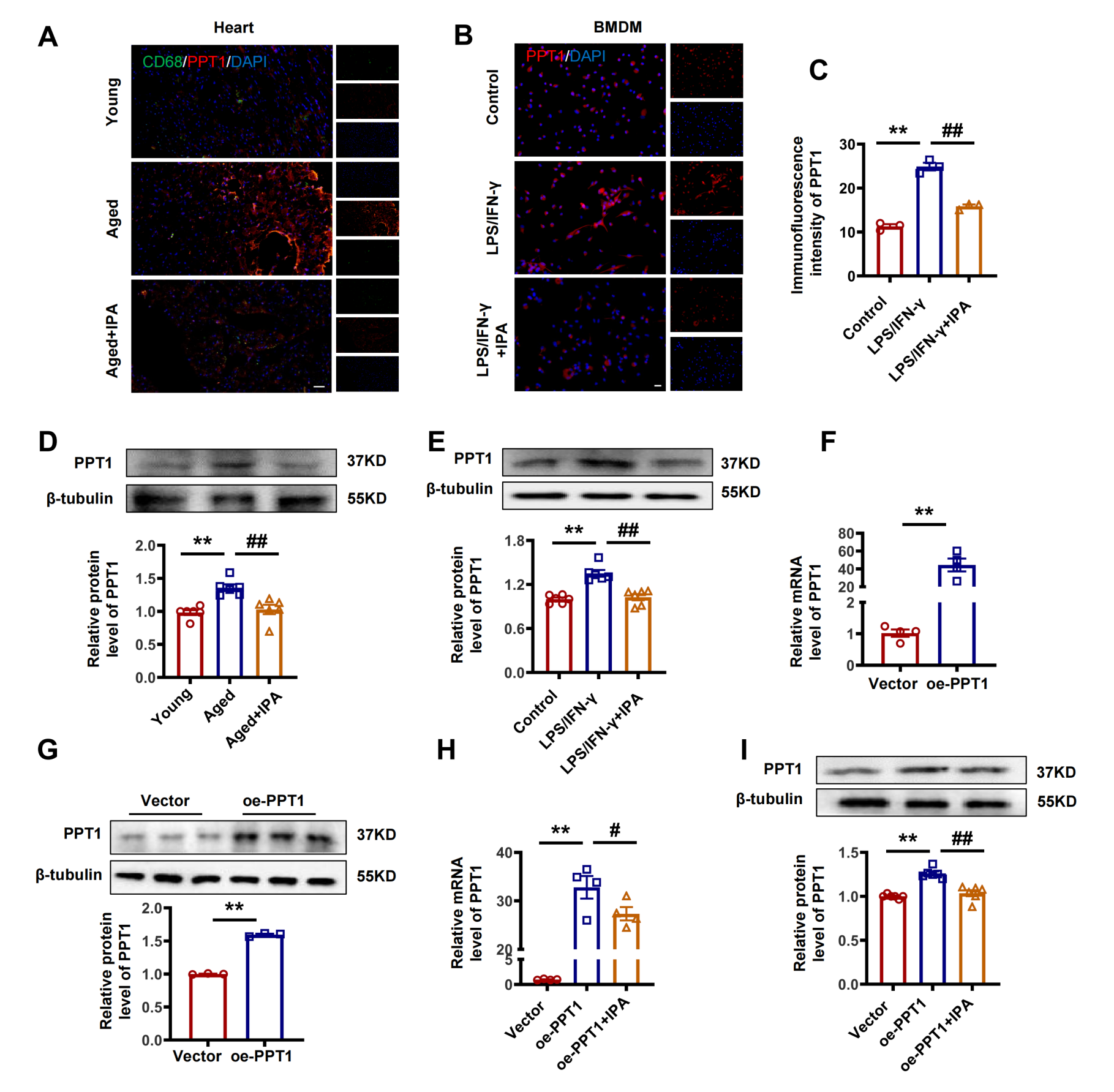


Supplementary Figure 7. IPA can inhibit the expression of PPT1 in macrophages.

**A)** Representative images of the immunofluorescence of CD68, PPT1, and DAPI in rat hearts. Magnification: 200×, scale bar=20 μm. **B**–**C)** Representative images and statistical graphs of the immunofluorescence of PPT1. Magnification: 200×, scale bar=20 μm. **D)** Representative figure and statistical analysis of PPT1 at protein level in heart. **E)** Representative figure and statistical analysis of PPT1 at protein level in M1 macrophage**. F)** Statistical chart for validation of PPT1 mRNA overexpression efficiency. **G)** Representative images and statistical analysis of PPT1 at protein level. **H**–**I)** Representative and statistical graphs of PPT1 both protein and mRNA level detection. (n = 3–6, data are expressed as mean ± SEM, ^**^*p* < 0.01 vs. the Young or Control or Vector group; ^#^*p* < 0.05, ^##^*p* < 0.01 vs. the Aged or LPS/IFN-γ or oe-PPT1 group)


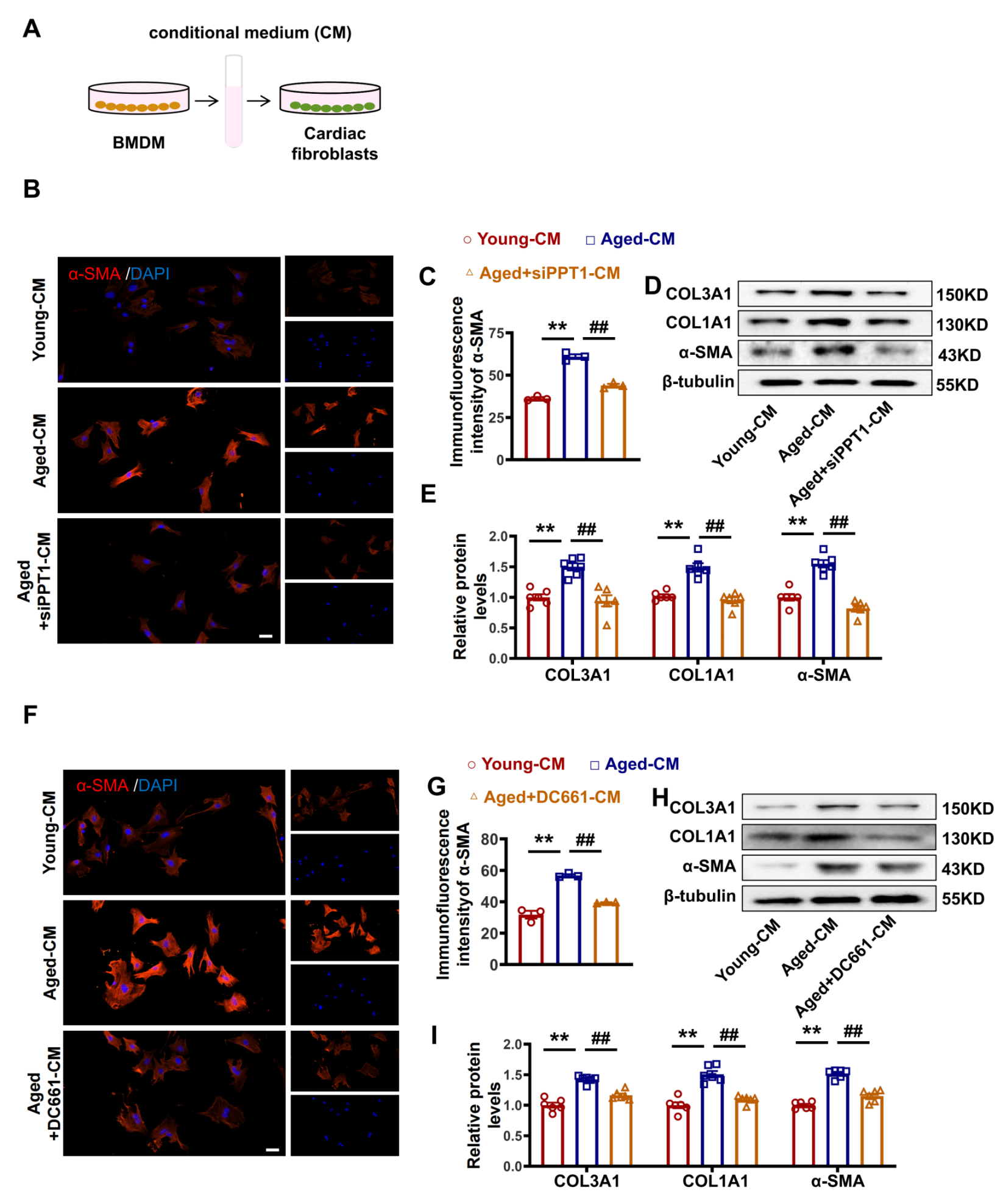


Supplementary Figure 8. Inhibition of PPT1 in aged macrophages reduces collagen deposition in fibroblasts

**A)** Schematic of the experimental design. **B**–**C)** Representative images and statistical graphs of the immunofluorescence of α-SMA. Magnification: 200×, scale bar=20 μm. **D**–**E)** Representative images and statistical analysis of COL3A1, COL1A1 and α-SMA at protein level. **F**–**G)** Representative images and statistical graphs of the immunofluorescence of α-SMA. Magnification: 200×, scale bar=20 μm. **H**–**I)** Representative images and statistical analysis of COL3A1, COL1A1 and α-SMA at protein level. (n = 3–6, data are expressed as mean ± SEM, ^**^*p* < 0.01 vs. the Young-CM group; ^##^*p* < 0.01 vs. the Aged-CM group)


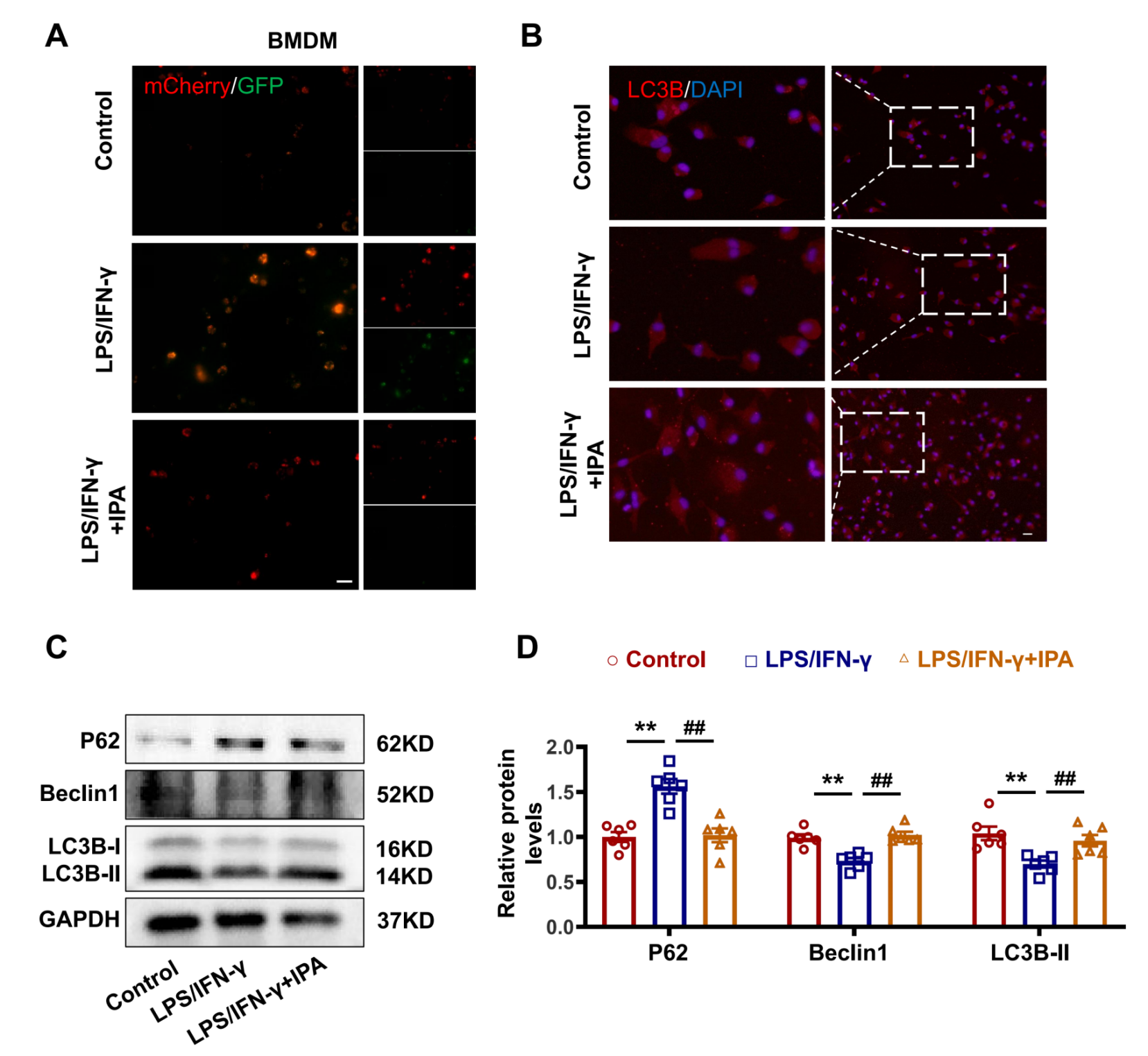


Supplementary Figure 9. IPA regulates autophagic activity of macrophages

**A)** mCherry-GFP-LC3B immunofluorescence staining representative images. Magnification: 400×, scale bar=20 μm. **B)** Representative images immunofluorescence staining of LC3B. Magnification: 200×, scale bar=20 μm. **C**–**D)** Representative images and statistical analysis of P62, Beclin1 and LC3B at protein level. (n =6, data are expressed as mean ± SEM, ^**^*p* < 0.01 vs. the Control group; ^##^*p* < 0.01 vs. the LPS/IFN-γ group)


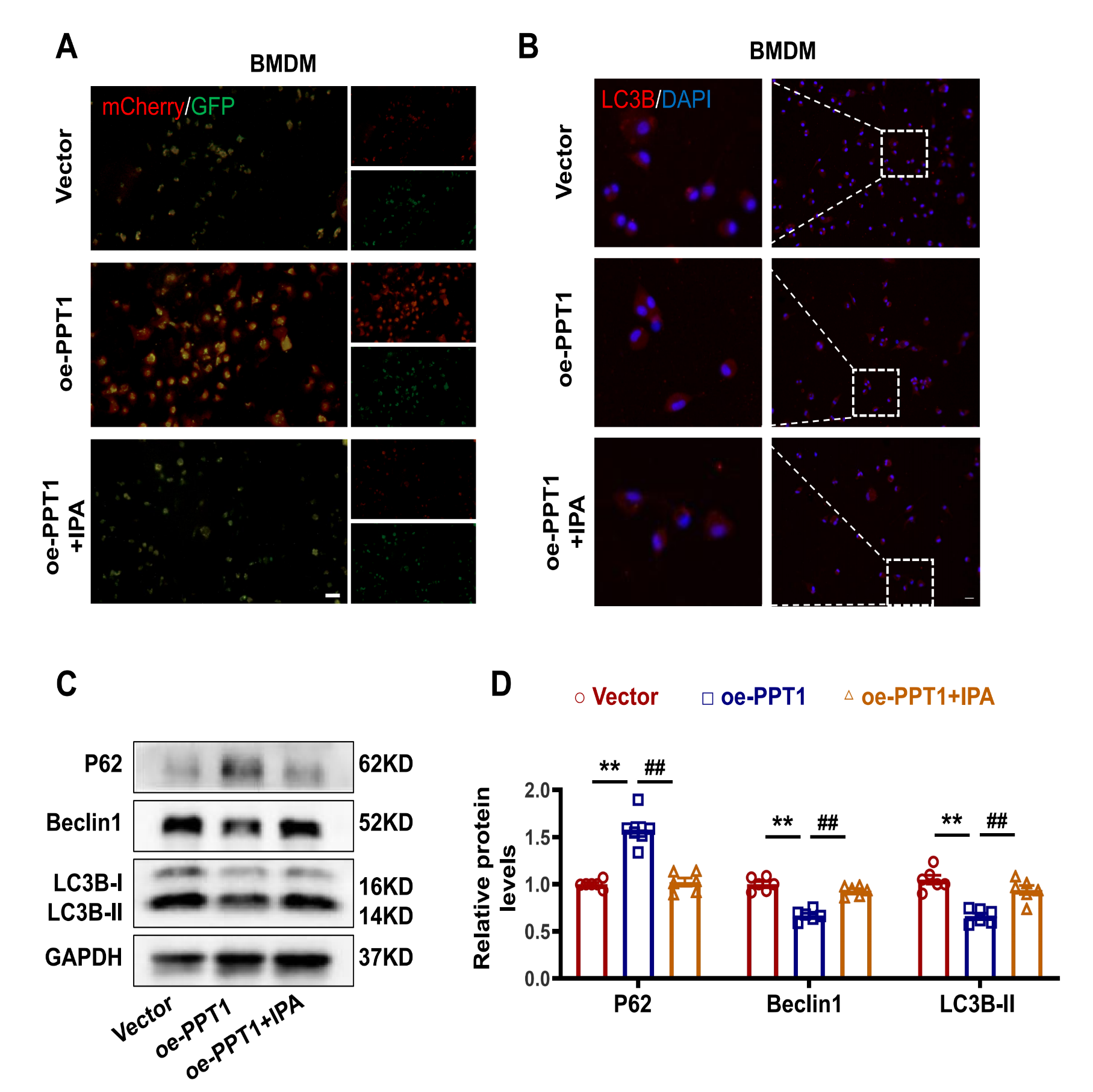


Supplementary Figure 10. IPA regulates PPT1 to affect autophagy activity

**A)** mCherry-GFP-LC3B immunofluorescence staining representative images. Magnification: 200×, scale bar=20 μm. **B)** Representative images immunofluorescence staining of LC3B. Magnification: 200×, scale bar=20 μm. **C**–**D)** Representative images and statistical analysis of P62, Beclin1 and LC3B at protein level. (n =6, data are expressed as mean ± SEM, ^**^*p* < 0.01 vs. the Vector group; ^##^*p* < 0.01 vs. the oe-PPT1 group)

Supplementary Table 1. Clinical characteristics of plasma samples donors

| Variable | Young  (n=14) | Older with non-HF  (n=30) | Older with HF  (n=30) | p-value |
| --- | --- | --- | --- | --- |
| Gender, m/f | 13/1 | 17/13 | 18/12 | 0.0495 |
| Age, y | 49 (44, 53) | 66.5 (62, 74) | 76 (65, 82) | <0.0001 |
| Smoking, n (%) | 9 (64.29) | 6 (20) | 15 (50) | 0.0081 |
| Drinking, n (%) | 6 (42.86) | 5 (16.67) | 6 (20) | 0.1386 |
| CAD, n (%) | 4 (28.57) | 17 (56.67) | 26 (86.67) | 0.0006 |
| Hypertension, n (%) | 5 (35.71) | 18 (60) | 18 (60) | 0.258 |
| Hyperlipidemia, n (%) | 7 (50) | 8 (26.67) | 2 (6.67) | 0.0052 |
| Diabetes n (%) | 4 (28.57) | 6 (20) | 10 (33.33) | 0.5033 |
| SBP, mmHg | 125 (110, 135) | 130 (120, 150) | 140 (120, 158) | 0.3719 |
| DBP, mmHg | 80 (70, 90) | 81 (80, 90) | 80 (70, 90) | 0.6265 |
| NT-proBNP, ng/L | 389.5 (16.79, 651.8) | 1016.6 (595.6, 1649) | 1542 (1076, 4851) | <0.0001 |

CAD, coronary artery disease; SBP, Systolic Blood Pressure; DBP, Diastolic Blood Pressure.
